# Exploring Machine Learning Applications in Pediatric Asthma Management: Scoping Review

**DOI:** 10.2196/57983

**Published:** 2024-08-27

**Authors:** Tanvi Ojha, Atushi Patel, Krishihan Sivapragasam, Radha Sharma, Tina Vosoughi, Becky Skidmore, Andrew D Pinto, Banafshe Hosseini

**Affiliations:** 1 Upstream Lab, MAP Centre for Urban Health Solutions Li Ka Shing Knowledge Institute St. Michael’s Hospital Toronto, ON Canada; 2 Temerty Faculty of Medicine University of Toronto Toronto, ON Canada; 3 Independent Information Specialist Ottawa, ON Canada; 4 Department of Family and Community Medicine St. Michael’s Hospital Toronto, ON Canada; 5 Department of Family and Community Medicine Temerty Faculty of Medicine University of Toronto Toronto, ON Canada; 6 Division of Clinical Public Health & Institute for Health Policy, Management and Evaluation Dalla Lana School of Public Health University of Toronto Toronto, ON Canada

**Keywords:** pediatric asthma, machine learning, predictive modeling, asthma management, exacerbation, artificial intelligence

## Abstract

**Background:**

The integration of machine learning (ML) in predicting asthma-related outcomes in children presents a novel approach in pediatric health care.

**Objective:**

This scoping review aims to analyze studies published since 2019, focusing on ML algorithms, their applications, and predictive performances.

**Methods:**

We searched Ovid MEDLINE ALL and Embase on Ovid, the Cochrane Library (Wiley), CINAHL (EBSCO), and Web of Science (core collection). The search covered the period from January 1, 2019, to July 18, 2023. Studies applying ML models in predicting asthma-related outcomes in children aged <18 years were included. Covidence was used for citation management, and the risk of bias was assessed using the Prediction Model Risk of Bias Assessment Tool.

**Results:**

From 1231 initial articles, 15 met our inclusion criteria. The sample size ranged from 74 to 87,413 patients. Most studies used multiple ML techniques, with logistic regression (n=7, 47%) and random forests (n=6, 40%) being the most common. Key outcomes included predicting asthma exacerbations, classifying asthma phenotypes, predicting asthma diagnoses, and identifying potential risk factors. For predicting exacerbations, recurrent neural networks and XGBoost showed high performance, with XGBoost achieving an area under the receiver operating characteristic curve (AUROC) of 0.76. In classifying asthma phenotypes, support vector machines were highly effective, achieving an AUROC of 0.79. For diagnosis prediction, artificial neural networks outperformed logistic regression, with an AUROC of 0.63. To identify risk factors focused on symptom severity and lung function, random forests achieved an AUROC of 0.88. Sound-based studies distinguished wheezing from nonwheezing and asthmatic from normal coughs. The risk of bias assessment revealed that most studies (n=8, 53%) exhibited low to moderate risk, ensuring a reasonable level of confidence in the findings. Common limitations across studies included data quality issues, sample size constraints, and interpretability concerns.

**Conclusions:**

This review highlights the diverse application of ML in predicting pediatric asthma outcomes, with each model offering unique strengths and challenges. Future research should address data quality, increase sample sizes, and enhance model interpretability to optimize ML utility in clinical settings for pediatric asthma management.

## Introduction

### Background

Asthma is characterized by inflammation and narrowing of the airways, leading to recurring episodes of wheezing, breathlessness, coughing, and chest tightness. As the most prevalent chronic childhood condition, asthma affects approximately 14% of children worldwide [1,2] and ranks among the top conditions for disability-adjusted life years in children [3]. Severe asthma exacerbations, defined as those requiring systemic corticosteroids, emergency department (ED) visits, or hospitalization, are not only the primary cause of urgent health care visits, hospitalizations, and asthma-related mortality in children but contribute to asthma-related morbidity and mortality in children, incurring substantial treatment costs [4,5].

Risk factors for asthma exacerbations are multifaceted, ranging from socioeconomic factors to environmental exposures. Low income, residing in areas of concentrated poverty, limited access to health care providers, and high medication costs are significant contributors [6-8]. In addition, factors such as systemic and interpersonal racial and ethnic discrimination, suboptimal asthma control, and environmental triggers play a crucial role in exacerbation development [9,10]. Specifically, aeroallergen exposure or sensitization and concurrent viral infections have been shown to significantly increase exacerbation risks [11-13]. Given this complex interplay of factors, accurately predicting severe asthma exacerbations in children remains a challenge. Accurate prediction of children at risk for severe exacerbations can facilitate preemptive care strategies, reduce morbidity, and enhance the quality of life of those affected [14].

Machine learning (ML), a branch of artificial intelligence (AI), emerges as a promising tool. A range of supervised learning techniques, such as linear and logistic regression, decision trees, and classifier methods, including support vector machines (SVMs) and gradient boosting, are used to predict specific data categories (eg, asthmatic vs nonasthmatic) or continuous variables (eg, lung function measurements) [15]. In contrast, unsupervised learning techniques, such as k-means clustering and hierarchical clustering, are used to develop models that enable the clustering of the data [15]. ML’s ability to analyze data and identify patterns has already shown success in various medical applications, including electrocardiography interpretation, heart failure classification, and diabetes outcome prediction [16-18]. In asthma management, AI has been instrumental in diagnosis, severity classification, and even in predicting asthma-related hospitalization risks at emergency encounters [19-22]. Several studies have investigated the role of AI in monitoring asthma exacerbations. Real-time assessment tools using environmental and physiological sensors have demonstrated notable accuracy in predicting exacerbations [23]. Contactless bed sensors for nocturnal data collection have also shown promise in detecting exacerbations [24]. In addition, AI-assisted clinical decision support tools, such as the Asthma Guidance and Prediction System, have been evaluated for their efficacy in reducing exacerbation frequency in children [25].

Recent advancements in ML offer promising tools for predicting asthma exacerbations. A previous systematic review highlighted the moderate predictive performance of traditional models, with emerging ML approaches showing potential for enhancing prediction accuracy [26]. Similarly, another recent systematic review and meta-analysis of 11 studies, focusing on participants aged ≥5 years with preexisting asthma diagnoses, demonstrated good discrimination. The overall pooled area under the receiver operating characteristic curve (AUROC) was 0.80 (95% CI 0.76-0.83), and the diagnostic odds ratio was 7.02 (95% CI 5.20-9.47), indicating that ML-based prediction models for asthma exacerbation could achieve substantial accuracy [27]. Notably, of the 11 studies included in the 2022 systematic review, 6 (55%) were conducted after 2019, indicating considerable advancements in a short period [27]. However, these studies focused on participants aged >5 years, leaving a gap in research for younger children [27]. Therefore, our scoping review aims to focus exclusively on studies conducted since 2019 that applied ML in predicting asthma exacerbations in children aged <18 years.

### Objectives

We intend to consolidate current knowledge by examining recent studies. This includes describing the types of predictive models developed, their applications in various settings, and the populations targeted and evaluating their performance in terms of accuracy, sensitivity, and specificity. This targeted approach will provide insights into the latest ML advancements and their potential to enhance pediatric asthma care.

## Methods

### Search Strategy

We registered this systematic review with PROSPERO (CRD42023440928) and have used the PRISMA-ScR (Preferred Reporting Items for Systematic Reviews and Meta-Analyses extension for Scoping Reviews) to guide our reporting.

### Search Strategy and Eligibility Criteria

An experienced information specialist (BS) developed and tested the search strategies in an iterative process in consultation with the review team. The MEDLINE strategy was peer reviewed by another senior information specialist before execution using the Peer Review of Electronic Search Strategies checklist [28]. Using the multifile and deduplication tool available on the Ovid platform, we searched Ovid MEDLINE ALL and Embase Classic+Embase. We also searched the Cochrane Library (Wiley), CINAHL (EBSCO), and Web of Science (core collection). All searches were performed on July 18, 2023. In addition, the reference lists of retrieved articles and relevant reviews were searched to identify other relevant studies.

The strategies used a combination of controlled vocabulary (eg, “Asthma,” “Artificial Intelligence,” and “Risk Assessment”) and keywords (eg, asthma, deep learning, and prognosis). There were no language restrictions on any of the searches, but results were limited to the publication years 2019 to the present. When possible, animal-only records, opinion pieces, and other irrelevant publication types (eg, case studies and conferences) were removed (refer to Multimedia Appendix 1 for strategies**)**. Records were downloaded and deduplicated using EndNote (version 9.3.3; Clarivate Analytics) and uploaded to Covidence (Veritas Health Innovation [29]) for efficient data management, extraction, and synthesis.

All studies were required to meet the eligibility criteria concerning the research focus, at both title/abstract and full-text screening: (1) in-vivo studies (human-based) that applied ML techniques to predict asthma-related outcomes, (2) participants aged <18 years, and (3) reported original data. The inclusion criteria were not limited to any specific study design to ensure inclusivity; hence, all available evidence from any study design was captured. There were no language restrictions for the studies reviewed. Studies were excluded if they were (1) in vitro studies (conducted on cellular substrates); (2) not focused on ML techniques to predict asthma-related outcomes; and (3) reviews, systematic reviews, opinions, editorials, and/or case reports.

### Data Collection

Covidence was used throughout the review to manage citations. We engaged and trained several individuals to assist with reviewing citations (AP, RS, TO, and TV). During both parts of the screening process, the reviewers used the eligibility criteria to evaluate and determine the inclusion or exclusion of studies, which were then reported in Covidence. The first-level screening consisted of title and abstract screening of all uploaded studies. Each citation was reviewed by 2 people independently to select studies for full-text review (RS and TO). If the eligibility criteria were met completely, as assessed by both reviewers, the studies were included. If studies did not meet eligibility criteria, as determined by both reviewers, they were excluded. Any citations in which there was a difference in opinion were brought to the study team to discuss, and a third reviewer decided on inclusion or exclusion (AP and TV). Second-level screening involved a thorough assessment of all the studies that passed the initial screening on the basis of their title and abstracts, performed independently by 2 reviewers (RS and TO). An additional second-level review was performed by a solo reviewer (AP), who excluded any studies that did not meet the same eligibility criteria in the primary step and were considered ineligible. The final set of studies included in this scoping review includes only those that passed the full-text screening process. Two members of the study team (RS and TO) independently assisted with data extraction, with each study being extracted once. Subsequently, a comparison check was performed on each extracted study by a third reviewer (AP).

The following data were extracted: authors, title, journal, publication year, funding source, ML application types, the intended purpose of ML application, identification of any potential bias in the ML model design (if applicable), bias mitigation strategies (if applicable), study design, research question/study objective, primary and secondary outcomes, country, demographics, sample size, youth age groups, the unit of analysis (individuals, groups, etc), data source (electronic medical records, databases, claims data, and health surveys), results, limitations, future research requirements (if applicable), use for clinical applications, and performance metrics (regression and classification). We noted if the information from an article was unavailable. A summary of the extracted information was recorded in Table S1 in Multimedia Appendix 2 [25,30-43].

### Risk of Bias Assessment

To assess the risk of bias, we used the Prediction Model Risk of Bias Assessment Tool (PROBAST) [44] and the guidelines for developing and reporting ML predictive models in biomedical research [45].

### Data Synthesis

In this review, we used a narrative synthesis to thoroughly review and summarize the objectives, ML algorithms, and clinical relevance of each study. We focused on how these studies used ML to predict asthma-related outcomes in children, detailing the different ML algorithms, such as random forests (RFs), logistic regression, and neural networks, that were used and how they were applied. We organized the studies using the ML techniques they used and gathered key performance measures, such as accuracy, sensitivity, and specificity for each one. We also noted studies that used >1 ML method and identified and documented common limitations found within the studies, such as small sample sizes and generalizability issues.

## Results

### Study Selection and Characteristics

**Figure 1:**
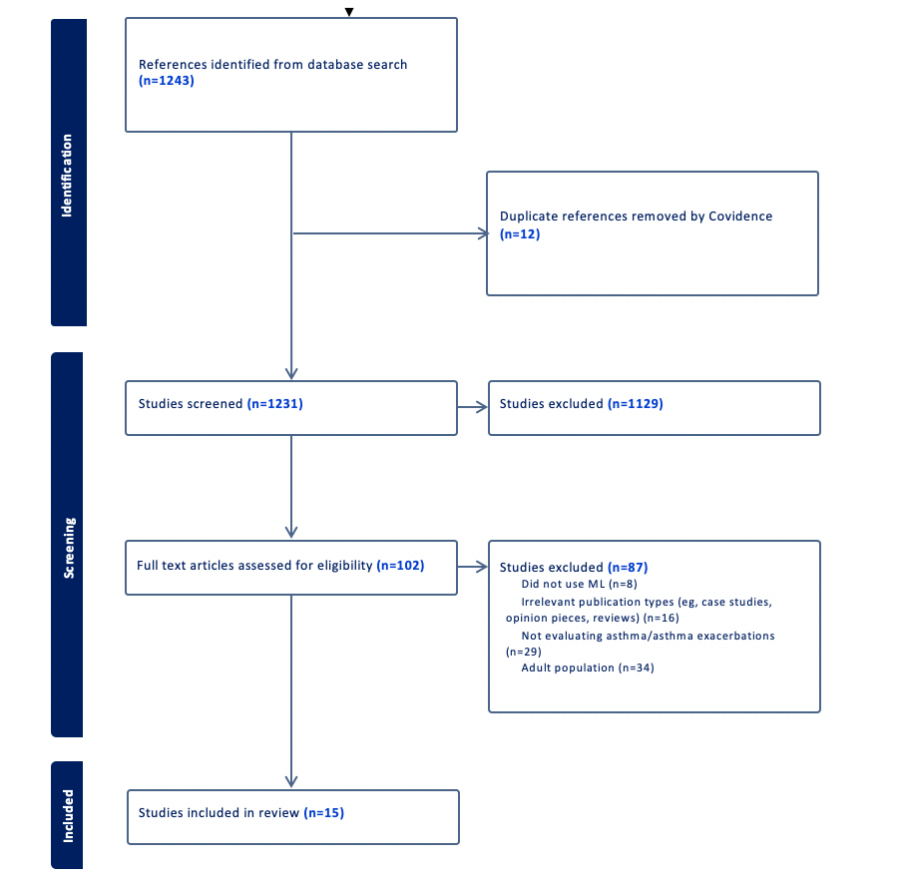
The selection process of eligible studies from all identified citations. ML: machine learning.

Our initial screening involved 1231 articles, from which 12 duplicates were removed using EndNote. This was followed by a primary screening that resulted in the inclusion of 102 studies. Upon secondary screening, 87 of these were excluded, leaving 15 articles that met our criteria for this review. The selection process is detailed in Figure 1.

The included studies, published between 2019 and 2023, predominantly came out in 2021 [25,30-43]. They originated from various countries, including the United States (n=10, 67%) [25,30,32,34,35,38,39,41-43], Germany (n=1, 7%) [40], New Zealand (n=1, 7%) [31], Japan (n=1, 7%) [36], the United Kingdom (n=1, 7%) [33], and Singapore (n=1, 7%) [37]. Sample sizes in these studies ranged from 74 to 87,413 pediatric patients, indicating a wide variation in the population sizes examined.

Table S1 in Multimedia Appendix 2 provides a comprehensive summary of the key data extracted from each included study. Most of these studies (n=9, 60%) implemented multiple ML techniques [30-34,38-40,43]. Logistic regression (n=7, 47%) and RFs (n=6, 40%) were the most commonly studied techniques [30-35,38-40,43]. This was followed by gradient boosting (n=4, 27%) [31,32,39,40] and artificial neural networks (ANNs; n=3, 20%) [30,38,41]. Decision trees (n=2, 13%) [34,36], natural language processing (NLP) models (n=2, 13%) [25,42], and Gaussian mixture models (n=1, 7%) [37] were the least frequent techniques used. Regarding study design, retrospective cohort studies were predominant (n=9, 60%) [30-32,35,38,39,41-43], with a smaller proportion being prospective cohorts (n=5, 33%) [33,34,36,37,40] and a single randomized controlled trial (n=1, 7%) [25]. Detailed information on the various ML models applied in the prediction of asthma exacerbations and related outcomes in children is provided in Tables S2-S8 in Multimedia Appendix 2.

### Quality Assessments

**Figure 2:**
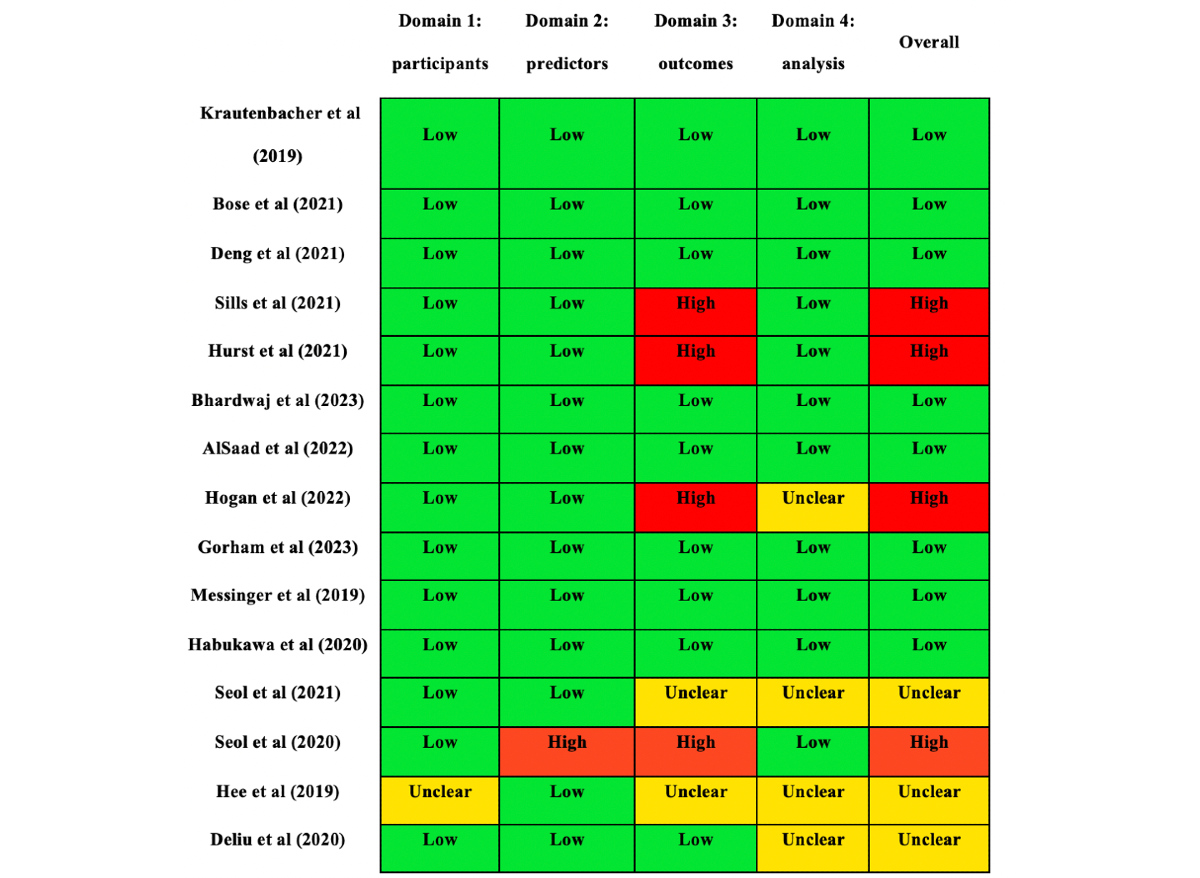
Risk of bias summary based on the Prediction Model Risk of Bias Assessment Tool quality assessment tool for included studies [25,30-43].

**Figure 3:**
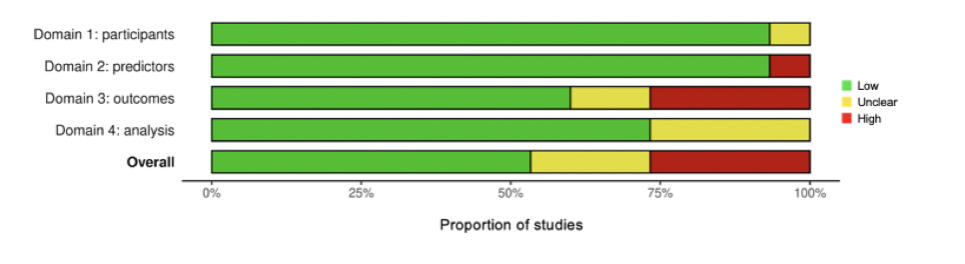
Summary of the risk of bias assessment.

The risk of bias in the included studies was assessed using the PROBAST tool [44]. Our analysis revealed that most studies (n=8, 53%) exhibited a low risk of bias [30-32,34-36,40,41], indicating robust methodologies and reporting. However, some studies (n=3, 20%) were classified with an unclear risk [33,37,42] because of insufficient detail in certain aspects, whereas a few studies (n=4, 27%) were identified as high risk [38,39,42,43], suggesting potential issues affecting their reliability. Studies classified as unclear or high risk often faced issues such as inconsistent definitions of outcomes across participants, outcome assessments influenced by prior knowledge of the predictors, or poorly specified inclusion and exclusion criteria for participants. Detailed breakdowns of each study’s bias assessment are presented in Figure 2, and a summary of the overall risk across all studies is depicted in Figure 3.

### ML Models in Pediatric Asthma: Predictive and Diagnostic Applications

Table 1 outlines the primary outcomes and the ML models used across the included studies. For predicting asthma exacerbations, the outcomes included any asthma-related health care encounter (outpatient visits, ED visits, and hospitalizations) or a prescription for a systemic steroid [25,30,35,38,39,43]. In classifying asthma phenotypes, the outcomes were the identification of allergic versus nonallergic asthma and the differentiation between mild and moderate-severe asthma [31,40,42]. For asthma diagnosis prediction, the outcomes were the prediction of an asthma diagnosis and the calculation of a pediatric asthma score (PAS) [32,41]. Studies identifying potential risk factors for asthma-related outcomes focused on outcomes, including the severity of symptoms and lung function, considering factors such as family history, medical history, and environmental triggers [33,34]. In sound-based diagnosis studies, the outcomes included the identification of wheezing versus nonwheezing sounds and the differentiation between asthmatic and normal coughs [36,37]. Features commonly used across studies include demographic data, such as sex, age, and race, despite significant variations in ML models and outcomes [25,30,35,38,39,43].

**Table 1:** Application of ML^a^ models in pediatric asthma management through predictive and diagnostic modalities.

Category	Outcome	Primary ML models
Prediction of asthma exacerbations [25,30,35,38,39,43]	Any encounter (outpatients, ED^b^ visits, and hospitalization) with an asthma-related *ICD-9* or *ICD-10*^c^ code or a prescription for a systemic steroid	Neural networks, LASSO^d^ regression, RFs^e^, XGBoost, and natural language processing
Classification of asthma phenotypes [31,40,42]	Allergic vs nonallergic asthma and mild vs moderate-severe asthma	SVMs^f^ and stochastic gradient boosting
Asthma diagnosis prediction [32,41]	Prediction of asthma diagnosis and PAS^g^	XGBoost, ANNs^h^, and natural language processing
Identification of potential risk factors for asthma [33,34]	Potential risk factors (such as family hx^i^, medical hx, and environmental triggers) for asthma-related outcomes (including symptom severity and lung function)	K-means clustering, RFs, and decision tree
Sound-based asthma or wheezing diagnosis [36,37]	Identification of wheezing vs nonwheezing sounds and differentiation between asthmatic and normal coughs	Decision trees and Gaussian mixture models

^a^ML: machine learning.

^b^ED: emergency department.

^c^ICD-9 or ICD-10: *International Classification of Diseases*, 9th or 10th revisions.

^d^LASSO: least absolute shrinkage and selection operator.

^e^RF: random forest.

^f^SVM: support vector machine.

^g^PAS: pediatric asthma score.

^h^ANN: artificial neural network.

^i^hx: history.

Table 2 provides a detailed summary of the predictors, clinical outcomes, and models used in the included studies. Studies have consistently used demographic data to predict asthma exacerbations. However, features related to medical history and health care use varied across the studies. Some studies focused on prescribed inhaled or oral steroids, previous health care use, and presence of moderate to severe asthma [25,30,35,39]. In contrast, others included variables such as time to triage, time to first medication and asthma medication, ED hourly volume, and patient disposition, including admitted or discharged [43]. Notably, some studies incorporated hospital characteristics, such as ownership (private vs public sector), teaching status, and size, along with family history factors such as alcohol or drug issues or housing instability [38]. Health insurance presence and type were also examined [39]. The models used in these studies included neural networks, least absolute shrinkage and selection operator regression, RFs, XGBoost, and NLP. The models were evaluated using metrics such as AUROC, accuracy, F_1_-score, precision, recall, and specific measures such as mean average negative predictive value (NPV). The best-performing models varied by application. Recurrent neural networks [30] and XGBoost showed high performance in predicting asthma exacerbations, with XGBoost achieving an AUROC of 0.761 [39]. ANNs outperformed logistic regression in predicting hospital readmissions, achieving an AUROC of 0.637 [38]. RFs were particularly effective in predicting hospitalization needs, with an AUROC of 0.886 [43].

A variety of demographics and clinical characteristics were used to differentiate between allergic and nonallergic asthma [31,40,42]. Key demographic variables included age, sex, weight, and race. Clinical parameters such as C-reactive protein levels, eosinophilic granulocytes, and oxygen saturation were also included in some studies [31]. Genetic markers, specifically protein kinase N2 and protein tyrosine kinase 2, along with breastfeeding duration, were also evaluated for their roles in asthma phenotypes [40]. In addition, some studies evaluated risk factors such as home conditions (eg, presence of carpets, home location and year, and animal triggers) and school characteristics, and home-related ventilators were considered to assess indoor environmental impacts on asthma [34]. ML models (eg, RFs, SVMs, gradient boosting, and decision trees) were used to analyze these variables. The most effective models varied across studies. Metrics such as AUROC, accuracy, precision, true positive rate, true negative rate, F_1_-score, prevalence ratios, and IQRs were used to evaluate the models’ performance. SVMs demonstrated high performance with metrics, including an accuracy of 77.8%, precision of 0.81, and an AUROC of 0.79. Stochastic gradient boosting achieved an AUROC of 0.81, highlighting its efficacy in incorporating genetic markers and breastfeeding duration.

**Table 2:** Summary of the included studies on ML^a^ applications in pediatric asthma: predictors, clinical outcomes, and models.

Study	Potential predictors, variables of interests-grouped	Metrics	Data source	Outcomes	ML models
AlSaad et al [30], 2022	Demographic data, medication use, health service use, clinical parameters and characteristics (comorbid illnesses), and insurance information	AUROC^b^ (0.85), AUC^c^-PR^d^ (0.74), and *F*_1_-score (0.61)	EHRs^e^	Frequency of ED^f^ use (number of visits made by pediatric patients during a 1-year predication window)	Deep learning: recurrent neural networks
Bhardwaj et al [31], 2023	Demographic data (age and weight) and clinical parameters and characteristics (C-reactive protein, eosinophilic granulocytes, oxygen saturation, premedication inhaled corticosteroid+long-acting β-2 agonist, other premedication, Pulmicort or celestamine during hospitalization, and azithromycin during hospitalization)	SVM^g^ differentiated between allergic and nonallergic asthma most well: accuracy (77.8%), precision (0.81), true positive rate (0.73), true negative rate (0.81), *F*_1_-score (0.81), and AUROC (0.79); because of the imbalance between both groups, a stratified 10-fold cross-validation was used	EHRs	Classify predominantly allergic asthma and nonallergic asthma among preschool children	RFs^h^, extreme gradient boosting, SVMs, adaptive boosting, extra tree classifier, and logistic regression
Bose et al [32], 2021	Demographic data (race, sex, ethnicity, and language spoken), geographic location (state of residency at the time of their first asthma diagnosis), insurance information (Medicaid enrollment), care site information (place of service such as EDs or office visits and provider specialties at first asthma diagnosis), medical hx^i^ (age of first and last asthma diagnoses and nonasthma-related clinical visits)	Mean ANSA, median ANSA, precision, recall, *F*_1_-score, and accuracy; XGBoost presented the best mean ANSA^j^: mean ANSA (0.43), median ANSA (0.43), precision (0.95), recall (0.82), *F*_1_-score (0.88), and accuracy (0.81)	EHRs	Occurrence of asthma diagnosis by the age of 10 years following an asthma incident	Naive Bayes, K-nearest neighbors, logistic regression, RFs, and XGBoost
Deliu et al [33], 2020	Medical hx and medication use (asthma diagnosis, use of asthma medication, current wheeze, asthma severity, and lung function) and risk factors (environmental tobacco smoke, pet ownership, length of breastfeeding, day-care attendance, presence of older siblings, and family hx of asthma)	FVC^k^, FEV1^l^, IE^m^, FE^n^ (early-onset frequent exacerbations), IE (93.7%), and FE (6.3%); shorter duration of breastfeeding was the strongest risk factor. FEV1/FVC of FE group: 85.1% at 8 years old	EHRs and health surveys	Examine risk factors that result in asthma-related outcomes in late childhood	K-means clustering
Deng et al [34], 2021	Demographic data (sex, race, age, and grade), family hx (job status, health status and hx, and education), insurance information, and risk factors (home conditions, such as carpet in house, tile flooring, or home location and year, animal triggers, home-related ventilators, and school characteristics)	Percentage and PR; top contributing factors: asthma, family rhinitis hx (relative importance: 10.40%), plant pollen trigger (relative importance: 5.48%), and bedroom carpet (relative importance: 3.58%). Allergy-related symptoms: plant pollen trigger (relative importance: 10.88%), higher paternal education (relative importance: 7.33%), and bedroom carpet (relative importance: 5.28%)	Health surveys	Evaluating factors in indoor environments (home vs school) contributing to asthma and allergy-related symptoms	RFs and decision tree
Gorham et al [35], 2023	Demographic data (age, sex, and race) and medical hx and medication use (inhaled or oral steroid prescribed, ED visits in a year, moderate to severe asthma, and asthma-related primary care visits in a year)	AUROC; internal validation: 0.769. 10-fold cross-validation AUROC: 0.737	EHRs	ED visit because of asthma exacerbations (also known as AER^o^); asthma exacerbations: asthma-related emergency	Logistic regression
Habukawa et al [36], 2020	Audio features (wheeze sounds: frequency, intensity, and duration) and demographic data (age)	Sensitivity, specificity, PPV^p^, and NPV^q^; sensitivity (100%), specificity (95.7%), PPV (90.3%), and NPV (100%)	EHRs	Identification of wheeze sounds vs nonwheeze sounds	Decision tree
Hee et al [37], 2019	Demographic data (age, sex, race, and weight), clinical parameters and characteristics (temperature, respiratory rate, heart rate, and shortness of breath), audio features (cough sounds: mel-frequency cepstral coefficients and constant-Q cepstral coefficients), and medical hx (asthma, allergic rhinitis, and recurrent wheeze)	Sensitivity (82.81%) and specificity (84.76%)	EHRs and health surveys	Classify and differentiate asthmatic coughs from normal voluntary coughs	Gaussian mixture model-universal background model
Hogan et al [38], 2022	Demographic data (sex and age), insurance, family hx (family member with alcohol or drug issues, hx of abuse, housing instability, and foster care), clinical parameters and characteristics (LOS^r^, admission season, and chronic conditions), and hospital characteristics (hospital ownership, teaching status, and hospital size)	AUC; logistic regression (0.592) and ANNs^s^ (0.637)	Claims data and biomedical databases	Asthma hospital readmission 180 days after hospital discharge	Logistic regression and ANNs
Hurst et al [39], 2022	Demographic data (age and sex), medical hx and medication use (comorbidities and prescribed asthma control plan), insurance, and health care use (inpatient admissions, ambulatory visits, and ED)	AUC at day 30, 90, and 180; LASSO^t^ (0.753, 0.740, and 0.732), RFs (0.757, 0.747, and 0.729), and XGBoost (0.761, 0.752, and 0.739)	EHRs and biomedical databases	Predict the occurrence of asthma exacerbation; asthma exacerbation: any encounter with an asthma-related ICD-9 or -10^u^ code and a prescription for a systemic steroid	LASSO, RFs, and XGBoost
Krautenbacher et al [40], 2019	Clinical parameters and characteristics (genes, including *PKN2*^v^, *PTK2*^w^, and *ALPP*^x^, and breastfeeding), and demographic data (age and sex)	AUC; boosting was the best model for all data sets: 0.81	Health surveys and biomedical databases	Distinguish between healthy children, those with mild to moderate allergic asthma, and those with nonallergic asthma	LASSO, elastic net, RFs, and stochastic gradient boosting
Messinger et al [41], 2019	Demographic data (age, sex, and race) and medication use, medical hx, and medications (LOS, PAS^y^ including vital sign data such as heart rate, respiratory rate, oxygen saturation, respiratory support, and medications)	Median absolute error; balanced set MAE^z^: 1.21	EHRs and biomedical databases	Use of vital sign data to predict the presence of asthma and to generate a novel pediatric-automated asthma score	ANNs
Seol et al [42], 2020	Demographic data (age, sex, ethnicity, and weight), family hx (asthma and smoking during pregnancy), medical hx (diagnosis of asthma, eczema, allergic rhinitis, eosinophilia, total IgE^aa^, asthma and associated outcomes such as persistent asthma, pertussis, pneumonia), and health care use (visits per year)	Percentage; NLP^ab^-PAC^ac^+/NLP–API^ad^+: 1614 (20%), NLP-PAC+ only: 954 (12%), NLP-API+ only: 105 (1%), and NLP-PAC–/NLP-API–: 5523 (67%); NLP-PAC) and NLP-API); asthmatic children classified as NLP-PAC+/NLP-API+ showed earlier onset asthma, more Th2^ae^-high profile, poorer lung function, higher asthma exacerbation, and higher risk of asthma-associated comorbidities compared with other groups	EHRs	Identifying characteristics that will identify childhood asthma and its subgroups using 2 algorithms	NLP
Seol et al [25], 2021	Medical hx and medications (IgE count, eosinophil count, smoking exposure, hx of allergic rhinitis, previous exacerbations, asthma diagnosis, and medication use) and demographic data (age, sex, and race)	IQR and *P* value; asthma exacerbation: intervention 12%, control 15%, *P*=.60; Time (min) taken by the clinician to take a clinical decision, median: intervention 3.5 min vs control 11.3 min	EHRs	Determine the presence of asthma exacerbation to reduce its frequency using clinical information; asthma exacerbation: ED visit, hospitalization, or outpatient visit requiring systemic corticosteroids for asthma	NLP
Sills et al [43], 2021	Demographic data (age, race, and sex), insurance, medical hx, and medications (ED and treatment factors: time to triage, time to first medication and asthma medication, ED hourly volume, and disposition including admitted or discharged)	AUC, accuracy, and *F*_1_-; model 1: triage (RF-AUC 0.831, accuracy 0.777, and *F*_1_-score 0.635, and logistic regression-AUC 0.795, accuracy 0.731, and *F*_1_-score 0.564); model 2: 60 minutes after patients’ arrival (RF-AUC 0.886, accuracy 0.795, and *F*_1_-score 0.689, and logistic regression-AUC 0.823, accuracy 0.753, and *F*_1_-score 0.618)	EHRs	Predict the need for hospitalization of pediatric patients with asthma	RFs and logistic regression

^a^ML: machine learning.

^b^AUROC: area under the receiver operating characteristic curve.

^c^AUC: area under cover.

^d^PR: precision recall.

^e^EHR: electronic health record.

^f^ED: emergency department.

^g^SVM: support vector machine.

^h^RF: random forest.

^i^hx: history.

^j^ANSA: average negative predictive value specificity area.

^k^FVC: forced vital capacity.

^l^FEV1: forced expiratory volume in the first second.

^m^IE: infrequent exacerbation.

^n^FE: frequent exacerbation.

^o^AER: asthma emergency risk.

^p^PPV: positive predictive value.

^q^NPV: negative predictive value.

^r^LOS: length of stay.

^s^ANN: artificial neural network.

^t^LASSO: least absolute shrinkage and selection operator.

^u^ICD-9 or -10: International Classification of Diseases, 9th or 10th Revisions.

^v^PKN2: protein kinase N2.

^w^PTK2: protein tyrosine kinase 2.

^x^ALPP: alkaline phosphatase, placental.

^y^PAS: pediatric asthma score.

^z^MAE: masked autoencoder.

^aa^IgE: immunoglobulin E.

^ab^NLP: natural language processing.

^ac^PAC: predetermined asthma criteria.

^ad^API: Asthma Predictive Index.

^ae^Th2: T helper 2 cells.

Studies that attempted to predict asthma diagnosis included a range of features, ML models, and metrics [32,41]. One study used demographic data such as race, sex, ethnicity, and language spoken, alongside medical history factors such as age at first and last asthma diagnoses and the number of nonasthma-related clinical visits, as well as geographic information such as the state of residency at the time of the first asthma diagnosis and insurance details, including Medicaid enrollment [32]. Another study focused on using patients’ medical history and medication use, along with vital sign data, to predict the presence of asthma and generate a novel PAS [41]. Various ML models were used, including naive Bayes, k-nearest neighbors, logistic regression, RFs, ANNs, and XGBoost, with ANNs and XGBoost showing the best performance. The metrics used to evaluate these models included mean average NPV specificity area, median average NPV specificity area, precision, recall, F_1_-score, and accuracy.

To identify potential risk factors for asthma-related outcomes, particularly focusing on the severity of symptoms and lung function, various ML models were used [33,34]. One study examined a range of variables, including medical history and medication use, such as asthma diagnosis, current wheeze, asthma severity, and lung function, alongside risk factors such as environmental tobacco smoke, pet ownership, length of breastfeeding, day-care attendance, presence of older siblings, and family history of asthma. K-means clustering was used to identify patterns and categorize risk factors associated with different asthma outcomes [33]. Evaluation metrics included forced vital capacity and forced expiratory volume in the first second, with specific attention to infrequent exacerbations and early-onset frequent exacerbations. Shorter breastfeeding duration emerged as the strongest risk factor, with the forced expiratory volume in the first second/forced vital capacity ratio in the frequent exacerbation group being 85.1% at 8 years old [33]. Another study focused on demographic data, such as sex, race, age, and grade, along with family history variables, including job status, health status, and education [34]. The study also considered insurance information and risk factors such as home conditions (eg, presence of carpets or tile flooring and home location and year), animal triggers, home-related ventilators, and school characteristics. Using RFs and decision trees, the study identified key contributors to asthma and allergy-related symptoms. The metrics used included prevalence ratios. Significant factors for asthma included a family history of rhinitis (relative importance of 10.40%), plant pollen trigger (relative importance of 5.48%), and bedroom carpet (relative importance of 3.58%). For allergy-related symptoms, important factors were plant pollen trigger (relative importance of 10.88%), higher paternal education (relative importance of 7.33%), and bedroom carpet (relative importance of 5.28%) [34].

To identify and classify asthmatic sounds, particularly focusing on wheezing and cough patterns, various ML models were used through a combination of audio features, demographic, and clinical data [36,37]. One study focused on differentiating between wheezing and nonwheezing sounds using a decision tree model [36]. The key features analyzed included audio characteristics such as the frequency, intensity, and duration of wheezing sounds, along with demographic data such as age. The model’s performance was evaluated using metrics such as sensitivity, specificity, positive predictive value, and NPV. The decision tree model achieved a sensitivity of 100%, specificity of 95.7%, positive predictive value of 90.3%, and NPV of 100%, demonstrating its high accuracy in identifying wheezing sounds among pediatric patients [36]. Another study aimed to classify and differentiate asthmatic coughs from normal voluntary coughs using a Gaussian mixture model-universal background model [37]. This study incorporated audio features such as mel-frequency cepstral coefficients and constant-Q cepstral coefficients, along with demographic data (age, sex, race, and weight) and clinical parameters (temperature, respiratory rate, heart rate, and shortness of breath). In addition, medical history factors such as asthma, allergic rhinitis, and recurrent wheezing were included. The model’s effectiveness was measured using sensitivity and specificity, achieving sensitivity of 82.81% and specificity of 84.76% [37]. These metrics indicate the model’s robustness in accurately classifying asthmatic coughs and distinguishing them from normal coughs.

### Common Limitations in the Reviewed Studies

A recurring theme in the limitations reported by the included studies pertains to challenges with data quality and completeness. Issues such as missing, incomplete, or limited data availability from medical records and health surveys were highlighted in several studies [34,38,41-43]. These data constraints can significantly impact the robustness and generalizability of the study findings. In the context of predicting asthma exacerbations, 3 studies specifically cited deficiencies in electronic health records (EHRs) [30,41,42] and pointed out the lack of critical variables in EHRs, such as socioeconomic status and adherence to treatment. These deficiencies arose from variables not being commonly recorded in EHRs. The absence of these variables can limit the depth and accuracy of predictive modeling, thereby affecting the models’ performance and generalizability. Another notable limitation was the issue of imbalanced data sets [30-32], which refers to situations where the number of observations in different classes is disproportionately distributed. For example, if there are significantly more cases of nonasthmatic patients compared to patients with asthma, this imbalance can lead to biased or skewed models that do not perform well across all classes. Small sample sizes, which can affect the statistical power and validity of the findings, were also a concern in a few studies [25,31,33,40]. A small sample size generally refers to a data set that is not large enough to yield statistically significant results or reliable conclusions. This can vary depending on the study design and statistical methods used, but typically, small sample sizes limit the ability to generalize findings to a larger population. In addition, limitations were identified in studies focusing on wheezing and asthmatic cough recognition algorithms. For example, a study developed a wheeze detection device for use in home environments, raising questions about its clinical value because of the specific context of its intended application [36]. Similarly, another study [37] on an asthmatic cough recognition algorithm highlighted that its validity and accuracy depended on the correct labeling of coughs by attending physicians. These limitations underscore the need for improved data quality and data collection processes to enhance the reliability and applicability of ML models in pediatric asthma research.

## Discussion

### Principal Findings

This scoping review successfully identified 15 peer-reviewed studies published since 2019, focusing on ML models in predicting pediatric asthma outcomes. Model use was diverse: logistic regression (7 studies), RFs (6 studies), gradient boosting (4 studies), ANNs (3 studies), decision trees (2 studies), NLP (2 studies), and Gaussian mixture model (1 study), with area under the curve ranging from 0.62 to 0.88. Most studies (n=8, 53%) had a low to moderate risk of bias, and they were evaluated using PROBAST.

### Comparative Analysis of ML Models

Among traditional ML models, logistic regression has demonstrated robustness, particularly in predicting hospitalization needs in pediatric asthma cases [30-33,35,38,43]. However, comparing logistic regression to RFs reveals that the latter offers superior performance in certain scenarios. For instance, RFs exhibited a higher area under the curve at the 1-hour postarrival time point in predicting hospitalization needs [43].

Gradient boosting models, particularly XGBoost, showed promise in certain scenarios. For example, in predicting early childhood asthma persistence, XGBoost matched the accuracy of logistic regression [32]. However, these models still lag slightly behind logistic regression and RFs in classifying asthma types, highlighting the potential differences in model efficacy across various applications.

The application of ANN provided promising results in predicting ED visits and asthma readmissions [30,38]. However, their performance, especially in complex clinical settings, warrants additional exploration and comparison with more conventional models. Decision trees, applied in more niche areas such as environmental risk assessment and wheeze sound recognition, demonstrated high accuracy and specificity [34,36]. NLP models, used within EHRs, helped early identification of pediatric asthma criteria [25,42], and Gaussian mixture models were applied to differentiate between patients with asthma and nonasthmatic patients through auditory recognition of types of coughs [37].

### Application of Predictive Models Across Different Outcomes

Among the 15 studies, key outcomes include predicting asthma exacerbations requiring urgent care, classifying asthma phenotypes by identifying allergic versus nonallergic asthma and severity levels, predicting asthma diagnoses and calculating PAS, and identifying potential risk factors such as symptom severity and lung function. In addition, sound-based diagnosis studies focused on distinguishing wheezing and differentiating asthmatic from normal coughs. One study [39] developed predictive models for pediatric asthma exacerbations using sociodemographic data, comorbidities, medication prescriptions, prescribed asthma controller plans, and patient service use history. This algorithm functioned as a potent tool capable of identifying children at risk of asthma exacerbations. Consequently, it signaled when preventive measures would be valuable to implement. Several studies used ML models to predict hospitalization needs and readmission risks using demographic variables. The studies by Sills et al [43] and Hogan et al [38] used ML models using varying features, including demographic variables such as sex, age, and race to predict hospitalization needs and readmission risks. Sills et al [43] demonstrated the potential of 2 distinct ML models to predict hospitalization in pediatric asthma cases, highlighting the models’ utility as supportive tools for clinical decision-making.

Similarly, Hogan et al [38] used an ANN algorithm to predict asthma readmissions within 180 days after discharge, finding that ANN outperformed traditional models in identifying readmission predictors. AlSaad et al [30] and Gorham et al [35] conducted studies focusing on predicting ED visits using data from EHRs/electronic medical records. Notably, the studies found that increased access to primary care with regular follow-ups resulted in fewer ED visits, suggesting that more frequent visits allowed for better assessment and management of asthma. Their findings suggest that ML models can effectively identify children with asthma who are at higher risk of repeated ED visits. Given the challenges associated with frequent ED use in emergency care, these prediction models emerge as valuable tools in enhancing asthma management and assisting in clinical decision-making.

We also examined the role of ML in asthma diagnosis in a pediatric population. One study [37] developed an ML model to distinguish between asthmatic and normal coughs by creating a database of cough sounds from asthmatic and nonasthmatic children. Another study [36] focused on an ML-based wheeze detection algorithm, analyzing lung sounds recorded through stethoscopes. Both these studies exemplify the use of ML in identifying asthma symptoms accurately. In addition, an ML algorithm was explored to automate asthma severity scoring, aiming to create a pediatric asthma respiratory score from vital sign data [41]. Additional research [42] used an NLP model to identify asthma early in children, and another study [25] developed the Asthma Guidance and Prediction System using ML and NLP to enhance asthma management programs and reduce asthma exacerbations. These studies collectively demonstrate the considerable potential of ML in improving the diagnosis, severity assessment, and management of pediatric asthma.

In examining asthma phenotypes, several studies have leveraged ML to categorize different characteristics of asthma. Two studies implemented various ML techniques [31,32], focusing on EHR data to classify asthma types. One study [31] aimed to distinguish between allergic and nonallergic asthma, whereas another study [32] sought to predict persistent versus transient asthma. Similarly, 2 studies [25,42] used EHR data and applied an NLP algorithm to identify pediatric asthma subgroups. This capability to distinguish between different types of asthma can significantly inform clinical decisions and guide parents in choosing appropriate asthma treatments, as highlighted by others [32].

Further support for the use of ML in understanding asthma phenotypes and allergies comes from the studies of Deng et al [34] and Krautenbacher et al [40], each adopting a unique approach. Deng et al [34] used ML models to assess risk factors in home and school environments affecting asthma and allergies. In contrast, Krautenbacher et al [40] developed a unique ML method to enhance the prediction of childhood asthma phenotypes, specifically distinguishing between allergic and nonallergic asthma, using various inputs such as genotypes, questionnaires, and diagnostic tools. Both studies effectively demonstrated the potential of ML models in identifying asthma and allergy risk factors as well as in improving the classification of childhood asthma types. Similarly, another study [33] applied ML to analyze wheeze exacerbation trajectories in children using medical record data, revealing diverse exacerbation patterns, early life risk factors, and asthma outcomes. This study aligns with the others in using ML to discern patterns predictive of childhood asthma. Jeddi et al [46] further emphasize the significance of these findings, noting that the ability to identify factors associated with childhood asthma via ML can help predict children considered susceptible. This prediction, in turn, enables the implementation of targeted interventions to prevent the onset of the disease.

### Future Directions and Key Considerations

Applying ML models to predict asthma outcomes in children involves several critical considerations to ensure accuracy, reliability, and applicability. The basis of any ML model is the data it is trained on. It should be comprehensive and include variables such as age, sex, family medical history, environmental exposures (such allergens, pollutants, and community viral loads), lifestyle factors (diet and physical activity), and clinical data (symptoms, medication use, lung function tests, etc). Several studies highlighted missing or incomplete data in medical records and health surveys [34,38,41-43], which underscores the importance of robust strategies for handling such data challenges. For example, studies have demonstrated that simple imputation methods, considering informative missingness, can be effective in managing missing numerical data in EHR for ML [47]. In addition, research on imputing missing values in laboratory data from EHRs has shown that the pattern of missingness is typically nonrandom and closely related to patients’ comorbidities, suggesting that multilevel imputation algorithms are more effective than cross-sectional methods [48].

Another point to consider is that asthma is a chronic condition with variable progression over time. Incorporating longitudinal data, which means tracking patient data over time, can help the model recognize patterns and predict future exacerbations or improvements. In addition, there is limited information on the choice of ML models across different age groups within the pediatric population. This gap highlights the need for future research to specifically address the performance and applicability of ML models in different pediatric age groups. This approach could provide valuable insights into age-specific predictive features and model adjustments.

Beyond accuracy, the model must also be interpretable [49]. Clinicians and patients should be able to understand how and why a particular prediction was made, which builds trust and ensures that the model’s findings are useful in real-world clinical decision-making. The model should also integrate seamlessly into existing clinical workflows. This involves considering how predictions will be delivered and their impact on clinical decision-making and ensuring they are in a format that health care providers can understand and easily incorporate into their existing decision-making processes. Previous research has shown that user-centered design is essential for successful implementation. For instance, a study involving 14 clinicians highlighted the need to identify patients at high risk and take proactive measures to manage asthma effectively [50]. Clinicians emphasized the importance of clear, actionable insights from the tool and understanding the underlying reasons for predictions. Barriers to implementation included usability and workflow integration challenges; the need for clear algorithm explainability; and ensuring the tool’s acceptability, adoption, and sustainability through proper design and training [50]. By involving clinicians in the design process, the tool was tailored to meet their needs, which underscores the importance of user-centered design in developing effective clinical decision support tools.

Strengths of this review included a comprehensive and systematic search across multiple databases, along with establishing clearly defined inclusion and exclusion criteria. The structured study selection process added robustness to the review. In addition, the use of the PROBAST tool for risk of bias assessment augmented the credibility of the review [44]. However, the review also had limitations that should be acknowledged. Despite a broad and inclusive search strategy designed to capture all subtypes of ML related to childhood asthma, some relevant studies might not be published in the indexed journals included in our search databases, and thus, there remains a possibility that some pertinent articles may have been inadvertently excluded.

This review highlights the potential of ML in transforming pediatric asthma care, from predicting exacerbations to characterizing asthma types. However, it also underscores the need for improved data quality, larger and more balanced data sets, and more rigorous validation to ensure these tools are clinically valuable. The exploration of varied ML techniques across studies offers a road map for future research to build more accurate, reliable, and applicable models for pediatric asthma management.

### Conclusions

This scoping review provides a broad overview of ML applications used to predict asthma-related outcomes in children. We reviewed a diverse range of studies focused on the design, training, testing, and interpretation of ML models and observed that using ML in childhood asthma is an emerging field that has seen significant growth over the past few years. This recent surge in research highlights the evolving nature and increasing interest in applying ML to improve pediatric asthma outcomes.

By leveraging data from multiple sources, ML approaches have made strides in identifying distinct asthma phenotypes, paving the way for more tailored and effective treatment strategies in clinical practice. However, the field faces ongoing challenges, particularly regarding minimizing missing data, ensuring robust model validation, and achieving interpretability. In addition, integrating these models smoothly into clinical workflows remains a key obstacle. While ML holds considerable promise in pediatric asthma research, the field is still evolving. To fully realize its potential, further research is needed to address these challenges and enhance the practical application of ML models in clinical settings.

## Appendix

Multimedia Appendix 1 Search strategy.

Multimedia Appendix 2 Study details.

Multimedia Appendix 3 PRISMA-ScR checklist.

## Abbreviations

AI: artificial intelligence
ANN: artificial neural network
AUROC: area under the receiver operating characteristic curve
ED: emergency department
EHR: electronic health record
ML: machine learning
NLP: natural language processing
NPV: negative predictive value
PRISMA-ScR: Preferred Reporting Items for Systematic Reviews and Meta-Analyses extension for Scoping Reviews
PROBAST: Prediction Model Risk of Bias Assessment Tool
RF: random forest
SVM: support vector machine

## Data Availability

The data sets generated and analyzed during this study are available from the corresponding author on reasonable request.
