# Supplementary material for: Exploring Machine Learning Applications in Pediatric Asthma Management: Scoping Review
Source: JMIR AI. 2024 Aug 27;3:e57983. doi: 10.2196/57983 (PMC11387921; doi:10.2196/57983)
Supplement: Multimedia Appendix 1 [file ai_v3i1e57983_app1.docx]

**Supplementary File 1. Full search strategy**

AI – Asthma in Children

Final Strategies

2023 Jul 18

Ovid Multifile

Database: Embase Classic+Embase <1947 to 2023 July 17>, Ovid MEDLINE(R) ALL <1946 to July 17, 2023>

Search Strategy:

--------------------------------------------------------------------------------

1 exp Asthma/ (461890)

2 (asthma* or lung allerg*).tw,kw,kf. (462731)

3 or/1-2 [ASTHMA] (561641)

4 exp Adult/ not (Adolescent/ or exp Child/ or exp Infant/) (15641223)

5 3 not 4 [ASTHMA - ADULT-ONLY REMOVED] (437302)

6 exp Artificial Intelligence/ (257784)

7 ((artificial or augmented or computer* or machine?) adj2 (heuristic* or intelligen* or learning* or neural network? or reasoning*)).tw,kw,kf. (293030)

8 AI.ti,kw,kf. (17155)

9 deep learning.tw,kw,kf. (106340)

10 expert system?.tw,kw,kf. (8160)

11 fuzzy logic.tw,kw,kf. (5901)

12 ((knowledge adj (base or bases)) or knowledgebase?).tw,kw,kf. (26292)

13 exp Neural Networks, Computer/ (150198)

14 (neural network* adj3 (automat* or model*)).tw,kw,kf. (31858)

15 perceptron?.tw,kw,kf. (9982)

16 Pattern Recognition, Automated/ (44097)

17 (pattern? adj3 recogni* adj5 (artificial* or automat* or comput* or machin*)).tw,kw,kf. (3219)

18 or/6-17 [AI Pt 1] (607321)

19 Data Mining/ (29044)

20 ((mine or mined or mines or mining) adj3 (data or text)).tw,kw,kf. (44526)

21 (datamin* or textmin*).tw,kw,kf. (610)

22 ((data or record?) adj3 (analys* or analytic* or linkage?)).tw,kw,kf. (897495)

23 or/19-22 [AI Pt 1] (946290)

24 18 or 23 [AI - Pts 1-2] (1518189)

25 5 and 24 [ASTHMA - AI/MACHINE LEARNING] (7796)

26 exp Risk Assessment/ (1045536)

27 (risk? adj3 (analys* or analyz* or analyt* or assess* or calculat* or estimat* or predict* or stratif*)).tw,kw,kf. (988749)

28 Risk Factors/ (1886239)

29 risk factor?.tw,kw,kf. (1914846)

30 (risk or risks).ti. (1481642)

31 ("Asthma-Guidance and Prediction System" or "A-GPS").tw,kw,kf. (1919)

32 Prognosis/ (1314368)

33 prognos*.tw,kw,kf. (2054712)

34 "Predictive Value of Tests"/ (393054)

35 predict*.tw,kw,kf. (4817145)

36 exp disease progression/ (390023)

37 ((asthma* or disease*) adj3 (deteriorat* or progress* or worse*)).tw,kw,kf. (582828)

38 exacerbat*.tw,kw,kf. (361412)

39 Models, Theoretical/ (235444)

40 exp Models, Statistical/ (1149350)

41 ((logistic* or statistical or theoretic*) adj2 (model* or regression?)).tw,kw,kf. (1164679)

42 model*.ti,kw,kf. (2001599)

43 exp Regression Analysis/ (994471)

44 (regression adj3 (analys#s or diagnostic*)).tw,kw,kf. (979090)

45 Clinical Decision-Making/ and (model* or rule or rules or tool or tools).tw,kw,kf. (20088)

46 Decision Support Techniques/ and (model* or rule or rules or tool or tools).tw,kw,kf. (24268)

47 Decision Support Systems, Clinical/ and (model* or rule or rules or tool or tools).tw,kw,kf. (7027)

48 Clinical Decision Rules/ (1590)

49 (decision* adj3 (aid or aids or algorithm* or model* or rule or rules or tool or tools)).tw,kw,kf. (106677)

50 or/26-49 [MODELS, PREDICTION, RISK, ETC] (13796176)

51 25 and 50 [ASTHMA - AI/MACHINE LEARNING - MODELS, PREDICTION, ETC] (4087)

52 exp Animals/ not Humans/ (17588120)

53 51 not 52 [ANIMAL-ONLY REMOVED] (3300)

54 (comment or editorial or news or newspaper article).pt. (2459027)

55 (letter not (letter and (Controlled Clinical Trial or Randomized Controlled Trial or Pragmatic Clinical Trial or Equivalence Trial or Observational Study or Comparative Study or Multicenter Study))).pt. (2468149)

56 53 not (54 or 55) [OPINION PIECES REMOVED] (3274)

57 (Case Reports not (Case Reports and (Controlled Clinical Trial or Randomized Controlled Trial or Pragmatic Clinical Trial or Equivalence Trial or Observational Study or Comparative Study or Multicenter Study or Systematic Review or Meta-Analysis))).pt. (2323015)

58 (case study or case studies or case reports?).ti. (161775)

59 56 not (57 or 58) [CASE-REPORTS ONLY REMOVED] (3261)

60 limit 59 to yr="2019-current" (1451)

61 60 use medall [MEDLINE RECORDS] (629)

62 exp asthma/ (461890)

63 (asthma* or lung allerg*).tw,kw,kf. (462731)

64 or/62-63 [ASTHMA] (561641)

65 exp adult/ not (exp adolescent/ or exp child/) (15738747)

66 64 not 65 [ASTHMA - ADULT-ONLY REMOVED] (436852)

67 exp artificial intelligence/ (257784)

68 ((artificial or augmented or computer* or machine?) adj2 (heuristic* or intelligen* or learning* or neural network? or reasoning*)).tw,kw,kf. (293030)

69 AI.ti,kw,kf. (17155)

70 deep learning/ (57375)

71 deep learning.tw,kw,kf. (106340)

72 machine learning/ (126416)

73 expert system/ (9305)

74 expert system?.tw,kw,kf. (8160)

75 fuzzy logic/ (10348)

76 fuzzy logic.tw,kw,kf. (5901)

77 ((knowledge adj (base or bases)) or knowledgebase?).tw,kw,kf. (26292)

78 exp artificial neural network/ (90593)

79 (neural network* adj3 (automat* or model*)).tw,kw,kf. (31858)

80 exp perceptron/ (63479)

81 perceptron?.tw,kw,kf. (9982)

82 automated pattern recognition/ (44097)

83 (pattern? adj3 recogni* adj5 (artificial* or automat* or comput* or machin*)).tw,kw,kf. (3219)

84 or/67-83 [AI Pt 1] (624949)

85 exp data mining/ (29920)

86 ((mine or mined or mines or mining) adj3 (data or text)).tw,kw,kf. (44526)

87 (datamin* or textmin*).tw,kw,kf. (610)

88 ((data or record?) adj3 (analys* or analytic* or linkage?)).tw,kw,kf. (897495)

89 or/85-88 [AI Pt 1] (947022)

90 84 or 89 [AI - Pts 1-2] (1535121)

91 66 and 90 [ASTHMA - AI/MACHINE LEARNING] (7902)

92 exp risk assessment/ (1045536)

93 (risk? adj3 (analys* or analyz* or analyt* or assess* or calculat* or estimat* or predict* or stratif*)).tw,kw,kf. (988749)

94 risk factor/ (2282619)

95 risk factor?.tw,kw,kf. (1914846)

96 (risk or risks).ti. (1481642)

97 ("Asthma-Guidance and Prediction System" or "A-GPS").tw,kw,kf. (1919)

98 prognosis/ (1314368)

99 prognos*.tw,kw,kf. (2054712)

100 prediction/ (503986)

101 predictive value/ (243453)

102 predict*.tw,kw,kf. (4817145)

103 exp disease exacerbation/ (390023)

104 ((asthma* or disease*) adj3 (deteriorat* or progress* or worse*)).tw,kw,kf. (582828)

105 exacerbat*.tw,kw,kf. (361412)

106 theoretical model/ (258905)

107 conceptual framework/ (37416)

108 exp statistical model/ (1149350)

109 ((logistic* or statistical or theoretic*) adj2 (model* or regression?)).tw,kw,kf. (1164679)

110 model*.ti,kw,kf. (2001599)

111 (regression adj3 (analys#s or diagnostic*)).tw,kw,kf. (979090)

112 clinical decision making/ and (model* or rule or rules or tool or tools).tw,kw,kf. (20088)

113 decision support system/ and (model* or rule or rules or tool or tools).tw,kw,kf. (13667)

114 clinical decision support system/ and (model* or rule or rules or tool or tools).tw,kw,kf. (7182)

115 clinical decision rule/ (1592)

116 (decision* adj3 (aid or aids or algorithm* or model* or rule or rules or tool or tools)).tw,kw,kf. (106677)

117 or/92-116 [MODELS, PREDICTION, RISK, ETC] (13841679)

118 91 and 117 [ASTHMA - AI/MACHINE LEARNING - MODELS, PREDICTION, ETC] (4158)

119 (exp animal/ or exp animal experimentation/ or exp animal model/ or exp animal experiment/ or nonhuman/ or exp vertebrate/) not (exp human/ or exp human experimentation/ or exp human experiment/) (13154344)

120 118 not 119 [ANIMAL-ONLY REMOVED] (4091)

121 editorial.pt. (1430938)

122 letter.pt. not (letter.pt. and (controlled clinical trial/ or exp randomized controlled trial/ or clinical trial/ or controlled study/ or cohort analysis/ or longitudinal study/ or prospective study/ or retrospective study/ or observational study/ or exp comparative study/)) (2304775)

123 120 not (121 or 122) [OPINION PIECES REMOVED] (4058)

124 exp case study/ not (exp case study/ and (controlled clinical trial/ or exp randomized controlled trial/ or clinical trial/ or controlled study/ or cohort analysis/ or longitudinal study/ or prospective study/ or retrospective study/ or observational study/ or exp comparative study/ or meta analysis/ or network meta-analysis/ or "systematic review"/)) (2355983)

125 (case study or case studies or case reports?).ti. (161775)

126 123 not (124 or 125) [CASE-REPORTS ONLY REMOVED] (4041)

127 conference abstract.pt. (4833106)

128 126 not 127 [CONFERENCE ABSTRACTS REMOVED] (3387)

129 limit 128 to yr="2019-current" (1315)

130 129 use emczd [EMBASE RECORDS] (687)

131 61 or 130 [BOTH DATABASES] (1316)

132 remove duplicates from 131 (930) [TOTAL UNIQUE RECORDS]

133 132 use medall [MEDLINE UNIQUE RECORDS] (622)

134 132 use emczd [EMBASE UNIQUE RECORDS] (308)

***************************

Cochrane Library

Date Run: 18/07/2023 16:09:37

ID Search Hits

#1 [mh Asthma] 14992

#2 (asthma* or (lung NEXT allerg*)):ti,ab,kw 37327

#3 #1 OR #2 37327

#4 [mh Adult] not ([mh Adolescent] or [mh Child] or [mh Infant]) 478107

#5 #3 NOT #4 31528

#6 [mh "Artificial Intelligence"] 2883

#7 ((artificial or augmented or computer* or machine or machines) NEAR/2 (heuristic* or intelligen* or learning* or "neural network" or "neural networks" or reasoning*)):ti,ab,kw 4489

#8 AI:ti 695

#9 "deep learning":ti,ab,kw 995

#10 (expert NEXT system*):ti,ab,kw 220

#11 "fuzzy logic":ti,ab,kw 73

#12 ((knowledge NEXT (base or bases)) or knowledgebase*):ti,ab,kw 542

#13 [mh "Neural Networks, Computer"] 529

#14 ((neural NEXT network*) NEAR/3 (automat* or model*)):ti,ab,kw 252

#15 perceptron*:ti,ab,kw 76

#16 [mh "Pattern Recognition, Automated"] 218

#17 (pattern* NEAR/3 recogni* NEAR/5 (artificial* or automat* or comput* or machin*)):ti,ab,kw 240

#18 {or #6-#17} 7780

#19 [mh ^"Data Mining"] 61

#20 ((mine or mined or mines or mining) NEAR/3 (data or text)):ti,ab,kw 241

#21 (datamin* or textmin*):ti,ab,kw 35

#22 ((data or record or records) NEAR/3 (analys* or analytic* or linkage or linkages)):ti,ab,kw 60734

#23 {or #19-#22} 60934

#24 #18 or #23 68244

#25 #5 AND #24 896

#26 [mh "Risk Assessment"] 13625

#27 ((risk or risks) NEAR/3 (analys* or analyz* or analyt* or assess* or calculat* or estimat* or predict* or stratif*)):ti,ab,kw 55111

#28 [mh ^"Risk Factors"] 32817

#29 ("risk factor" or "risk factors"):ti,ab,kw 82640

#30 (risk or risks):ti 50548

#31 ("Asthma-Guidance and Prediction System" or "A-GPS"):ti,ab,kw 50

#32 [mh Prognosis] 202495

#33 prognos*:ti,ab,kw 54055

#34 [mh "Predictive Value of Tests"] 8757

#35 predict*:ti,ab,kw 116462

#36 [mh "Disease Progression"] 9428

#37 ((asthma* or disease*) NEAR/3 (deteriorat* or progress* or worse*)):ti,ab,kw 37843

#38 exacerbat*:ti,ab,kw 23062

#39 [mh ^"Models, Theoretical"] 1005

#40 [mh "Models, Statistical"] 20118

#41 ((logistic* or statistical or theoretic*) NEAR/2 (model* or regression*)):ti,ab,kw 37322

#42 model*:ti 15090

#43 [mh "Regression Analysis"] 24423

#44 (regression NEAR/3 (analys* or diagnostic*)):ti,ab,kw 36195

#45 [mh "Clinical Decision-Making"] and (model* or rule or rules or tool or tools):ti,ab,kw 199

#46 [mh "Decision Support Techniques"] and (model* or rule or rules or tool or tools):ti,ab,kw 1531

#47 [mh "Decision Support Systems, Clinical"] and (model* or rule or rules or tool or tools):ti,ab,kw 246

#48 [mh "Clinical Decision Rules"] 43

#49 (decision* NEAR/3 (aid or aids or algorithm* or model* or rule or rules or tool or tools)):ti,ab,kw 5088

#50 {or #26-#49} 495472

#51 #25 and #50 523

#52 Conference Proceeding:pt 224784

#53 #51 NOT #52 421

#54 #51 NOT #52 with Cochrane Library publication date Between Jan 2019 and Dec 2023 175

CDSR – 34 reviews

Trials - 141

CINAHL

| # | Query | Limiters/Expanders | Last Run Via | Results |
| --- | --- | --- | --- | --- |
| S56 | S52 not (S53 or S54) | Limiters - Published Date: 20190101-20231231  Expanders - Apply equivalent subjects  Search modes - Boolean/Phrase | Interface - EBSCOhost Research Databases  Search Screen - Advanced Search  Database - CINAHL Plus with Full Text | 165 |
| S55 | S52 not (S53 or S54) | Expanders - Apply equivalent subjects  Search modes - Boolean/Phrase | Interface - EBSCOhost Research Databases  Search Screen - Advanced Search  Database - CINAHL Plus with Full Text | 482 |
| S54 | TI "case study" or "case studies" or "case reports" | Expanders - Apply equivalent subjects  Search modes - Boolean/Phrase | Interface - EBSCOhost Research Databases  Search Screen - Advanced Search  Database - CINAHL Plus with Full Text | 28,749 |
| S53 | PT case study | Expanders - Apply equivalent subjects  Search modes - Boolean/Phrase | Interface - EBSCOhost Research Databases  Search Screen - Advanced Search  Database - CINAHL Plus with Full Text | 474,504 |
| S52 | S50 NOT S51 | Expanders - Apply equivalent subjects  Search modes - Boolean/Phrase | Interface - EBSCOhost Research Databases  Search Screen - Advanced Search  Database - CINAHL Plus with Full Text | 487 |
| S51 | PT editorial or letter | Expanders - Apply equivalent subjects  Search modes - Boolean/Phrase | Interface - EBSCOhost Research Databases  Search Screen - Advanced Search  Database - CINAHL Plus with Full Text | 719,471 |
| S50 | S24 AND S49 | Expanders - Apply equivalent subjects  Search modes - Boolean/Phrase | Interface - EBSCOhost Research Databases  Search Screen - Advanced Search  Database - CINAHL Plus with Full Text | 488 |
| S49 | S25 OR S26 OR S27 OR S28 OR S29 OR S30 OR S31 OR S32 OR S33 OR S34 OR S35 OR S36 OR S37 OR S38 OR S39 OR S40 OR S41 OR S42 OR S43 OR S46 OR S47 OR S48 | Expanders - Apply equivalent subjects  Search modes - Boolean/Phrase | Interface - EBSCOhost Research Databases  Search Screen - Advanced Search  Database - CINAHL Plus with Full Text | 1,589,371 |
| S48 | TI ( decision* N3 (aid or aids or algorithm* or model* or rule or rules or tool or tools) ) OR AB ( decision* N3 (aid or aids or algorithm* or model* or rule or rules or tool or tools) ) | Expanders - Apply equivalent subjects  Search modes - Boolean/Phrase | Interface - EBSCOhost Research Databases  Search Screen - Advanced Search  Database - CINAHL Plus with Full Text | 17,581 |
| S47 | (MH "Clinical Prediction Rules") | Expanders - Apply equivalent subjects  Search modes - Boolean/Phrase | Interface - EBSCOhost Research Databases  Search Screen - Advanced Search  Database - CINAHL Plus with Full Text | 50 |
| S46 | S44 AND S45 | Expanders - Apply equivalent subjects  Search modes - Boolean/Phrase | Interface - EBSCOhost Research Databases  Search Screen - Advanced Search  Database - CINAHL Plus with Full Text | 14,127 |
| S45 | TI ( model* or rule or rules or tool or tools ) OR AB ( model* or rule or rules or tool or tools ) | Expanders - Apply equivalent subjects  Search modes - Boolean/Phrase | Interface - EBSCOhost Research Databases  Search Screen - Advanced Search  Database - CINAHL Plus with Full Text | 868,222 |
| S44 | (MH "Decision Making, Clinical+") OR (MH "Decision Support Techniques+") OR (MH "Decision Support Systems, Clinical") | Expanders - Apply equivalent subjects  Search modes - Boolean/Phrase | Interface - EBSCOhost Research Databases  Search Screen - Advanced Search  Database - CINAHL Plus with Full Text | 55,554 |
| S43 | TI ( regression N3 (analys* or diagnostic*) ) OR AB ( regression N3 (analys* or diagnostic*) ) | Expanders - Apply equivalent subjects  Search modes - Boolean/Phrase | Interface - EBSCOhost Research Databases  Search Screen - Advanced Search  Database - CINAHL Plus with Full Text | 134,811 |
| S42 | (MH "Regression+") | Expanders - Apply equivalent subjects  Search modes - Boolean/Phrase | Interface - EBSCOhost Research Databases  Search Screen - Advanced Search  Database - CINAHL Plus with Full Text | 332,082 |
| S41 | TI model* | Expanders - Apply equivalent subjects  Search modes - Boolean/Phrase | Interface - EBSCOhost Research Databases  Search Screen - Advanced Search  Database - CINAHL Plus with Full Text | 132,658 |
| S40 | TI ( (logistic* or statistical or theoretic*) N2 (model* or regression*) ) OR AB ( (logistic* or statistical or theoretic*) N2 (model* or regression*) ) | Expanders - Apply equivalent subjects  Search modes - Boolean/Phrase | Interface - EBSCOhost Research Databases  Search Screen - Advanced Search  Database - CINAHL Plus with Full Text | 165,354 |
| S39 | (MH "Models, Statistical+") | Expanders - Apply equivalent subjects  Search modes - Boolean/Phrase | Interface - EBSCOhost Research Databases  Search Screen - Advanced Search  Database - CINAHL Plus with Full Text | 41,755 |
| S38 | (MH "Models, Theoretical") | Expanders - Apply equivalent subjects  Search modes - Boolean/Phrase | Interface - EBSCOhost Research Databases  Search Screen - Advanced Search  Database - CINAHL Plus with Full Text | 50,158 |
| S37 | TI exacerbat* OR AB exacerbat* | Expanders - Apply equivalent subjects  Search modes - Boolean/Phrase | Interface - EBSCOhost Research Databases  Search Screen - Advanced Search  Database - CINAHL Plus with Full Text | 31,456 |
| S36 | TI ( (asthma* or disease*) N3 (deteriorat* or progress* or worse*) ) OR AB ( (asthma* or disease*) N3 (deteriorat* or progress* or worse*) ) | Expanders - Apply equivalent subjects  Search modes - Boolean/Phrase | Interface - EBSCOhost Research Databases  Search Screen - Advanced Search  Database - CINAHL Plus with Full Text | 44,505 |
| S35 | (MH "Disease Progression+") | Expanders - Apply equivalent subjects  Search modes - Boolean/Phrase | Interface - EBSCOhost Research Databases  Search Screen - Advanced Search  Database - CINAHL Plus with Full Text | 56,425 |
| S34 | TI predict* OR AB predict* | Expanders - Apply equivalent subjects  Search modes - Boolean/Phrase | Interface - EBSCOhost Research Databases  Search Screen - Advanced Search  Database - CINAHL Plus with Full Text | 461,186 |
| S33 | (MH "Predictive Value of Tests") | Expanders - Apply equivalent subjects  Search modes - Boolean/Phrase | Interface - EBSCOhost Research Databases  Search Screen - Advanced Search  Database - CINAHL Plus with Full Text | 57,575 |
| S32 | TI prognos* OR AB prognos* | Expanders - Apply equivalent subjects  Search modes - Boolean/Phrase | Interface - EBSCOhost Research Databases  Search Screen - Advanced Search  Database - CINAHL Plus with Full Text | 146,366 |
| S31 | (MH "Prognosis") | Expanders - Apply equivalent subjects  Search modes - Boolean/Phrase | Interface - EBSCOhost Research Databases  Search Screen - Advanced Search  Database - CINAHL Plus with Full Text | 95,518 |
| S30 | TI ( "Asthma-Guidance and Prediction System" or "A-GPS" ) OR AB ( "Asthma-Guidance and Prediction System" or "A-GPS" ) | Expanders - Apply equivalent subjects  Search modes - Boolean/Phrase | Interface - EBSCOhost Research Databases  Search Screen - Advanced Search  Database - CINAHL Plus with Full Text | 177 |
| S29 | TI risk or risks | Expanders - Apply equivalent subjects  Search modes - Boolean/Phrase | Interface - EBSCOhost Research Databases  Search Screen - Advanced Search  Database - CINAHL Plus with Full Text | 266,592 |
| S28 | TI ( "risk factor" or "risk factors" ) OR AB ( "risk factor" or "risk factors" ) | Expanders - Apply equivalent subjects  Search modes - Boolean/Phrase | Interface - EBSCOhost Research Databases  Search Screen - Advanced Search  Database - CINAHL Plus with Full Text | 224,809 |
| S27 | (MH "Risk Factors") | Expanders - Apply equivalent subjects  Search modes - Boolean/Phrase | Interface - EBSCOhost Research Databases  Search Screen - Advanced Search  Database - CINAHL Plus with Full Text | 199,859 |
| S26 | TI ( (risk or risks) N3 (analys* or analyz* or analyt* or assess* or calculat* or estimat* or predict* or stratif*) ) OR AB ( (risk or risks) N3 (analys* or analyz* or analyt* or assess* or calculat* or estimat* or predict* or stratif*) ) | Expanders - Apply equivalent subjects  Search modes - Boolean/Phrase | Interface - EBSCOhost Research Databases  Search Screen - Advanced Search  Database - CINAHL Plus with Full Text | 131,806 |
| S25 | (MH "Risk Assessment") | Expanders - Apply equivalent subjects  Search modes - Boolean/Phrase | Interface - EBSCOhost Research Databases  Search Screen - Advanced Search  Database - CINAHL Plus with Full Text | 149,487 |
| S24 | S5 AND S23 | Expanders - Apply equivalent subjects  Search modes - Boolean/Phrase | Interface - EBSCOhost Research Databases  Search Screen - Advanced Search  Database - CINAHL Plus with Full Text | 920 |
| S23 | S17 OR S22 | Expanders - Apply equivalent subjects  Search modes - Boolean/Phrase | Interface - EBSCOhost Research Databases  Search Screen - Advanced Search  Database - CINAHL Plus with Full Text | 195,956 |
| S22 | S18 OR S19 OR S20 OR S21 | Expanders - Apply equivalent subjects  Search modes - Boolean/Phrase | Interface - EBSCOhost Research Databases  Search Screen - Advanced Search  Database - CINAHL Plus with Full Text | 148,041 |
| S21 | TI ( (data or record or records) N3 (analys* or analytic* or linkage or linkages) ) OR AB ( (data or record or records) N3 (analys* or analytic* or linkage or linkages) ) | Expanders - Apply equivalent subjects  Search modes - Boolean/Phrase | Interface - EBSCOhost Research Databases  Search Screen - Advanced Search  Database - CINAHL Plus with Full Text | 142,900 |
| S20 | TI ( datamin* or textmin* ) OR AB ( datamin* or textmin* ) | Expanders - Apply equivalent subjects  Search modes - Boolean/Phrase | Interface - EBSCOhost Research Databases  Search Screen - Advanced Search  Database - CINAHL Plus with Full Text | 29 |
| S19 | TI ( (mine or mined or mines or mining) N3 (data or text) ) OR AB ( (mine or mined or mines or mining) N3 (data or text) ) | Expanders - Apply equivalent subjects  Search modes - Boolean/Phrase | Interface - EBSCOhost Research Databases  Search Screen - Advanced Search  Database - CINAHL Plus with Full Text | 3,338 |
| S18 | (MH "Data Mining") | Expanders - Apply equivalent subjects  Search modes - Boolean/Phrase | Interface - EBSCOhost Research Databases  Search Screen - Advanced Search  Database - CINAHL Plus with Full Text | 4,298 |
| S17 | S6 OR S7 OR S8 OR S9 OR S10 OR S11 OR S12 OR S13 OR S14 OR S15 OR S16 | Expanders - Apply equivalent subjects  Search modes - Boolean/Phrase | Interface - EBSCOhost Research Databases  Search Screen - Advanced Search  Database - CINAHL Plus with Full Text | 51,108 |
| S16 | TI ( pattern* N3 recogni* N5 (artificial* or automat* or comput* or machin*) ) OR AB ( pattern* N3 recogni* N5 (artificial* or automat* or comput* or machin*) ) | Expanders - Apply equivalent subjects  Search modes - Boolean/Phrase | Interface - EBSCOhost Research Databases  Search Screen - Advanced Search  Database - CINAHL Plus with Full Text | 149 |
| S15 | TI perceptron* OR AB perceptron* | Expanders - Apply equivalent subjects  Search modes - Boolean/Phrase | Interface - EBSCOhost Research Databases  Search Screen - Advanced Search  Database - CINAHL Plus with Full Text | 345 |
| S14 | TI ( ((neural W0 network*) N3 (automat* or model*)) ) OR AB ( ((neural W0 network*) N3 (automat* or model*)) ) | Expanders - Apply equivalent subjects  Search modes - Boolean/Phrase | Interface - EBSCOhost Research Databases  Search Screen - Advanced Search  Database - CINAHL Plus with Full Text | 1,367 |
| S13 | (MH "Neural Networks (Computer)") | Expanders - Apply equivalent subjects  Search modes - Boolean/Phrase | Interface - EBSCOhost Research Databases  Search Screen - Advanced Search  Database - CINAHL Plus with Full Text | 3,847 |
| S12 | TI ( (knowledge W0 (base or bases)) or knowledgebase* ) OR AB ( (knowledge W0 (base or bases)) or knowledgebase* ) | Expanders - Apply equivalent subjects  Search modes - Boolean/Phrase | Interface - EBSCOhost Research Databases  Search Screen - Advanced Search  Database - CINAHL Plus with Full Text | 4,891 |
| S11 | TI "fuzzy logic" OR AB "fuzzy logic" | Expanders - Apply equivalent subjects  Search modes - Boolean/Phrase | Interface - EBSCOhost Research Databases  Search Screen - Advanced Search  Database - CINAHL Plus with Full Text | 281 |
| S10 | TI expert W0 system# OR AB expert W0 system# | Expanders - Apply equivalent subjects  Search modes - Boolean/Phrase | Interface - EBSCOhost Research Databases  Search Screen - Advanced Search  Database - CINAHL Plus with Full Text | 506 |
| S9 | TI "deep learning" OR AB "deep learning" | Expanders - Apply equivalent subjects  Search modes - Boolean/Phrase | Interface - EBSCOhost Research Databases  Search Screen - Advanced Search  Database - CINAHL Plus with Full Text | 5,338 |
| S8 | TI AI | Expanders - Apply equivalent subjects  Search modes - Boolean/Phrase | Interface - EBSCOhost Research Databases  Search Screen - Advanced Search  Database - CINAHL Plus with Full Text | 2,449 |
| S7 | TI ( (artificial or augmented or computer* or machine or machines) N2 (heuristic* or intelligen* or learning* or "neural network" or "neural networks" or reasoning*) ) OR AB ( (artificial or augmented or computer* or machine or machines) N2 (heuristic* or intelligen* or learning* or "neural network" or "neural networks" or reasoning*) ) | Expanders - Apply equivalent subjects  Search modes - Boolean/Phrase | Interface - EBSCOhost Research Databases  Search Screen - Advanced Search  Database - CINAHL Plus with Full Text | 22,157 |
| S6 | (MH "Artificial Intelligence+") | Expanders - Apply equivalent subjects  Search modes - Boolean/Phrase | Interface - EBSCOhost Research Databases  Search Screen - Advanced Search  Database - CINAHL Plus with Full Text | 29,704 |
| S5 | S3 NOT S4 | Expanders - Apply equivalent subjects  Search modes - Boolean/Phrase | Interface - EBSCOhost Research Databases  Search Screen - Advanced Search  Database - CINAHL Plus with Full Text | 42,571 |
| S4 | (MH "Adult+") NOT (MH "Child+" or MH "Adolescence+") | Expanders - Apply equivalent subjects  Search modes - Boolean/Phrase | Interface - EBSCOhost Research Databases  Search Screen - Advanced Search  Database - CINAHL Plus with Full Text | 1,635,203 |
| S3 | S1 OR S2 | Expanders - Apply equivalent subjects  Search modes - Boolean/Phrase | Interface - EBSCOhost Research Databases  Search Screen - Advanced Search  Database - CINAHL Plus with Full Text | 50,477 |
| S2 | TI ( asthma* or (lung W0 allerg*) ) OR AB ( asthma* or (lung W0 allerg*) ) | Expanders - Apply equivalent subjects  Search modes - Boolean/Phrase | Interface - EBSCOhost Research Databases  Search Screen - Advanced Search  Database - CINAHL Plus with Full Text | 42,728 |
| S1 | (MH "Asthma+") | Expanders - Apply equivalent subjects  Search modes - Boolean/Phrase | Interface - EBSCOhost Research Databases  Search Screen - Advanced Search  Database - CINAHL Plus with Full Text | 37,986 |

Web of Science

| Set # | Search Query | Results |
| --- | --- | --- |
| 1 | asthma* or (lung NEAR/0 allerg*) (Topic) | 241167 |
| 2 | TS=((artificial or augmented or computer* or machine or machines) NEAR/2 (heuristic* or intelligen* or learning* or "neural network" or "neural networks" or reasoning*) ) OR TS=("deep learning" ) OR TS=("expert system" or "expert systems") OR TS=("fuzzy logic" ) OR TS=((knowledge NEAR/0 (base or bases)) or knowledgebase* ) OR TI=(AI) | 885055 |
| 3 | ("neural network" or "neural networks") NEAR/3 (automat* or model*) (Topic) OR perceptron* (Topic) OR pattern* NEAR/3 recogni* NEAR/5 (artificial* or automat* or comput* or machin*) (Topic) | 136098 |
| 4 | (mine or mined or mines or mining) NEAR/3 (data or text) (Topic) OR datamin* or textmin* (Topic) OR (data or record or records) NEAR/3 (analys* or analytic* or linkage or linkages) (Topic) | 1051086 |
| 5 | #2 OR #3 OR #4 | 1937810 |
| 6 | #1 AND #5 | 5118 |
| 7 | (risk or risks) NEAR/3 (analys* or analyz* or analyt* or assess* or calculat* or estimat* or predict* or stratif*) (Topic) OR "risk factor" or "risk factors" (Topic) OR risk or risks (Title) | 2119634 |
| 8 | "Asthma-Guidance and Prediction System" or "A-GPS" (Topic) OR prognos* (Topic) OR predict* (Topic) | 5171346 |
| 9 | (asthma* or disease*) NEAR/3 (deteriorat* or progress* or worse*) (Topic) OR exacerbat* (Topic) | 417622 |
| 10 | (logistic* or statistical or theoretic*) NEAR/2 (model* or regression*) (Topic) OR model* (Title) OR regression NEAR/3 (analys* or diagnostic*) (Topic) | 3969387 |
| 11 | decision* NEAR/3 (aid or aids or algorithm* or model* or rule or rules or tool or tools) (Topic) | 161331 |
| 12 | #7 OR #8 OR #9 OR #10 OR #11 | 10075346 |
| 13 | #12 AND #6 | 2744 |
| 14 | DT=(Book OR Book Chapter OR Book Review OR Editorial Material OR Letter OR Meeting OR Meeting Abstract OR Meeting Summary) | 22112853 |
| 15 | (#12 AND #6) NOT DT=(Book OR Book Chapter OR Book Review OR Editorial Material OR Letter OR Meeting OR Meeting Abstract OR Meeting Summary) | 2681 |
| 16 | "case study" or "case studies" or "case reports" (Title) | 307445 |
| 17 | (#12 AND #6) NOT (DT=(Book OR Book Chapter OR Book Review OR Editorial Material OR Letter OR Meeting OR Meeting Abstract OR Meeting Summary) OR TI=("case study" or "case studies" or "case reports")) | 2667 |
| 18 | (#12 AND #6) NOT (DT=(Book OR Book Chapter OR Book Review OR Editorial Material OR Letter OR Meeting OR Meeting Abstract OR Meeting Summary) OR TI=("case study" or "case studies" or "case reports")) and 2023 or 2022 or 2021 or 2019 or 2020 (Publication Years) | 1149 |
| 19 | child or children or baby or babies or infant or infants or toddler or toddlers or schoolage or "school-aged" or adolescen* or preadolescen* or "pre-adolescence" or "pre-adolescents" or teen or teens or teenage* or youth or youths or pediatric* or paediatric* (Topic) | 3,139,764 |
| 20 | #19 AND #20 | 414 |
