# Supplementary material for: Exploring Machine Learning Applications in Pediatric Asthma Management: Scoping Review
Source: JMIR AI. 2024 Aug 27;3:e57983. doi: 10.2196/57983 (PMC11387921; doi:10.2196/57983)
Supplement: Multimedia Appendix 2 [file ai_v3i1e57983_app2.docx]

**Supplementary Table 1 (Table S1)**

| **Study** | **Title** | **Journal** | **Country** | **Study Design** | **Age Group** | **ML Techniques** | **Data Source** | **Sample Size** | **Features** | **ML/Study Objective** | **Results** |
| --- | --- | --- | --- | --- | --- | --- | --- | --- | --- | --- | --- |
| Krautenbacher  2019[40] | A strategy for high-dimensional multivariable analysis classifies childhood asthma phenotypes from genetic, immunological, and environmental factors | Allergy: EAACI | Germany | Prospective Cohort | 4-14 years | Stochastic gradient boosting; Random Forest (RF); Lasso, elastic net | Databases (Biomedical); Health Surveys | 260 | 7 modalities | To predict different childhood asthma phenotypes (healthy children, mild‐to‐moderate allergic asthmatics, and nonallergic asthmatic) based off an assembled questionnaire, diagnostic, genotype, microarray, RT‐qPCR, flow cytometry, and cytokine data as inputs | SG AUC for performed prediction on single modalities separately: 0.54 SG Overall AUC for complete observations including annotated genes: 0.77  SG Overall AUC for complete observations including non-annotated genes: 0.81  RF AUC for performed prediction on single modalities separately: 0.66  RF Overall AUC for complete observations including annotated genes: 0.7  RF Overall AUC for complete observations including non-annotated genes: 0.7 LASSO AUC for performed prediction on single modalities separately: 0.69 LASSO Overall AUC for complete observations including annotated genes: 0.77  LASSO Overall AUC for complete observations including non-annotated genes: 0.77 |
| Bose  2021[32] | Personalized prediction of early childhood asthma persistence: A machine learning approach | PLoS ONE | United States of America | Retrospective Cohort | 2-5 years | Gradient boosting & AdaBoosting; Random Forest (RF); K-Nearest Neighbors (KNN); Naive Bayes ; Logistic Regression | EMRs | 9934 | 648 | To predict to predict whether a subsequent asthma diagnosis will occur by age 10 years (individual asthma persistence), given clinical input for a child under the age of 5 years with an incident asthma diagnosis | RF: Mean ANSA: 0.42, Median ANSA: 0.44, NPV: 0.33, Precision: 0.95, Recall: 0.82, F1 score: 0.88, Accuracy: 0.88 XGB: Mean ANSA: 0.43, Median ANSA: 0.43, NPV: 0.34, Precision: 0.95, Recall: 0.82, F1 score: 0.88, Accuracy: 0.81 Naive Bayes: Mean ANSA 0.29, Median ANSA 0.27, NPV 0.25, Precision 0.95, Recall 0.72, F1 score 0.82, Accuracy 0.72  K-nearest Neighbours: Mean ANSA 0.40, Median ANSA 0.41, NPV 0.29, Precision 0.95, Recall 0.78, F1 score 0.85, Accuracy 0.77 LR: Mean ANSA 0.42, Median ANSA 0.42, NPV 0.32, Precision 0.95, Recall 0.81, F1 score 0.88, Accuracy 0.81 |
| Deng  2021[34] | Application of data science methods to identify school and home risk factors for asthma and allergy-related symptoms among children in New York | The Science of the total environment | United States of America | Prospective Cohort | 8-14 years | Random Forest (RF); Decision Tree | Health Surveys | 74 | 84 | To use machine-learning methods to evaluate how indoor environmental conditions at home and school contribute to asthma and allergy-related symptoms | The top contributing factors identified for asthma were family rhinitis history, plant pollen trigger, bedroom carpet, environmental tobacco smoke (ETS) trigger symptom, and ETS exposure  For allergy-related symptoms, plant pollen trigger, higher paternal education, bedroom carpet, family rhinitis history, and higher maternal education were the strongest contributing factors primary heating with hot water radiator was negatively associated with asthma symptoms |
| Sills 2021[43] | Predicting hospitalization of pediatric asthma patients in emergency departments using machine learning | International Journal of Medical Informatics | United States of America | Retrospective Cohort | 4-10 years | Random Forest (RF); Logistic Regression; H2O Automl) | EMRs | 9,069 ED visits | 13 | To predict the need for hospitalization of pediatric asthma cases using two different ML models; one with data only available at ED triage, the other adding information available one hour into the ED visit. | RF Triage timepoint AUC: 0.831 RF Triage timepoint accuracy: 0.777 RF Triage timepoint F1 score: 0.635  RF 60 minutes post arrival AUC: 0.886 RF 60 minutes post arrival accuracy: 0.795 RF 60 minutes post arrival F1 score: 0.689 LR Triage timepoint AUC: 0.795 LR Triage timepoint accuracy: 0.731 LR Triage timepoint F1 score: 0.564 LR 60 minutes post arrival AUC: 0.823 LR 60 minutes post arrival accuracy: 0.753 LR 60 minutes post arrival F1 score: 0.618 automl Triage timepoint AUC: 0.914 automl Triage timepoint accuracy: 0.849 automl Triage timepoint F1 score: 0.789 automl 60 minutes post arrival AUC: 0.942 automl 60 minutes post arrival accuracy: 0.890 automl 60 minutes post arrival F1 score: 0.848 |
| Hurst  2021[39] | Environmental and clinical data utility in pediatric asthma exacerbation risk prediction models | BMC Medical Informatics and Decision Making | United States of America | Retrospective Cohort | 5-18 years | Linear regression; Gradient boosting & AdaBoosting; Random Forest (RF) | EMRs; Databases (Biomedical) | 5982 | 12 | To predict whether a child will have an asthma related exacerbation in the forthcoming 30-180 days. | RF AUC of overall model of exacerbation in 30 days: 0.757 RF AUC of overall model of exacerbation in 90 days: 0.747 RF AUC of overall model of exacerbation in 180 days: 0.729 XGB AUC of overall model of exacerbation in 30 days: 0.761 XGB AUC of overall model of exacerbation in 90 days: 0.752 XGB AUC of overall model of exacerbation in 180 days: 0.739 LASSO AUC of overall model of exacerbation in 30 days: 0.753 LASSO AUC of overall model of exacerbation in 90 days: 0.740 LASSO AUC of overall model of exacerbation in 180 days: 0.732 |
| Bhardwaj 2023[31] | Machine learning model for classification of predominantly allergic and non-allergic asthma among preschool children with asthma hospitalization | Journal of Asthma | New Zealand | Retrospective Cohort | 1-5 years | Random Forest (RF); Support Vector Machines (SVM); Logistic Regression; XGB | EMRs | 205 | 93 | To classify two forms of asthma in preschool children (predominantly allergic asthma and non-allergic asthma) | - Randrom Forest: Accuracy: 67.3%, Precision: 0.71, TPR 0.59, TNR: 0.72, F1: 0.72, ROC-AUC: 0.72  - XGB: Accuracy: 66.3%, Precision: 0.69, TPR 0.54, TNR: 0.75, F1: 0.71, ROC-AUC: 0.71 - AdaBoost:Accuracy: 63.4%, Precision: 0.70, TPR 0.62, TNR: 0.64, F1: 0.66, ROC-AUC: 0.66 - LR: Accuracy: 76.2%, Precision: 0.81, TPR 0.74, TNR: 0.78, F1: 0.79, ROC-AUC: 0.80 - SVM-Linear: Accuracy: 77.8%, Precision: 0.81, TPR 0.73, TNR: 0.81, F1: 0.81, ROC-AUC: 0.79 - ETC: Accuracy 69.6%, Precision: 0.74, TPR 0.63, TNR: 0.74, F1: 0.73, ROC-AUC: 0.71 |
| AlSaad 2022[30] | Predicting emergency department utilization among children with asthma using deep learning models | Healthcare Analytics | United States of America | Retrospective Cohort | 0-18 years | Three recurrent neural network (RNN) models: bidirectional long short-term memory (BiLSTM), bidirectional gated recurrent unit (BiGRU) and reverse time attention model (RETAIN) vs. baseline multinomial logistic regression model | EMRs | 87413 | Unspecified | To predict the frequency of pediatric asthma related emergency department visits in the next 12 months | - AUC-ROC: multinominal regression; micro 0.81, macro 0.66, RETAIN; micro 0.85. macro 0.72, BiLSTM; micro 0.85, macro 0.71, BiGRU; micro 0.85, macro 0.71  - AUC-PR: multinominal regression; micro 0.69, macro 0.43, RETAIN; micro 0.74, macro 0.44, BiLSTM; micro 0.74 macro 0.48, BiGRU; micro 0.74, macro 0.46  - F1 score: multinominal regression; micro 0.63, macro 0.56, RETAIN; micro 0.66, macro 0.60, BiLSTM; micro 0.67 macro 0.60, BiGRU; micro 0.66, macro 0.61 6 |
| Hogan 2022[38] | Comparing Artificial Intelligence and Traditional Methods to Identify Factors Associated With Pediatric Asthma Readmission | Academic Pediatrics | United States of America | Retrospective Cohort | 5-18 years | (Artificial) Neural Networks (ANN); Logistic Regression | Databases (Biomedical); Claims Data | 18489 | 18 | To predict whether a child has an asthma readmission within 180 days from hospitalization discharge | Overall model AUC of LR: 0.592 AUC in training set of ANN 0.637 AUC in validation set of ANN: 0.636 |
| Gorham 2023[35] | Predicting emergency department visits among children with asthma in two academic medical systems | Journal of Asthma | United States of America | Retrospective Cohort | 2-18 years | Logistic Regression | EMRs | 26008 | 8 | To predict which patients will visit the ED due to asthma exacerbation within one year of a primary care encounter | AUROC during model training: 0.768  AUROC against 2018 NCH internal validation: 0.769  AUROC against EU population without local retraining: 0.684 AUROC against EU population with local retraining: 0.737 |
| Messinger 2019[41] | Novel pediatric-automated respiratory score using physiologic data and machine learning in asthma | Pediatric Pulmonology | United States of America | Retrospective Cohort | 2-18 years | (Artificial) Neural Networks (ANN) | EMRs; Databases (Biomedical) | 128 | 3 | To create pediatric‐automated asthma respiratory score (pARS) by using the manual pediatric asthma score (PAS) as the clinical care standard | MAE of cascaded ANN with eight hidden layers trained with the balanced group: 1.21 |
| Habukawa 2020[36] | A wheeze recognition algorithm for practical implementation in children | PLOS ONE | Japan | Prospective Cohort | 0-13 years | Decision Tree | Health Surveys | 813 recorded sounds from 214 children | 5 | Automatic wheeze sound recognition | PPV 90.3%  NPPV 100%  Sensitivity (wheeze sounds): 100%  Sensitivity (non-wheeze sounds): 95.7% |
| Seol 2021[25] | Artificial intelligence-assisted clinical decision support for childhood asthma management: A randomized clinical trial | PLOS ONE | United States of America | Randomized Control Trial | 0-17 years | Language processing (NLP) algorithms | Unspecified (for applied ML technique) | 184 | Unspecified | To assess the effectiveness and efficiency of Asthma-Guidance and Prediction System (AGPS), an Artificial Intelligence (AI)-assisted CDS tool, in optimizing asthma management and reducing exacerbation frequency through a randomized clinical trial (RCT). | - The proportion of children with AE in both groups decreased from the baseline (P = 0.042), specifically 17% to12% in the intervention vs. 25% to 15% in the control group  - there was no difference in AE frequency between the two groups (12% for the intervention group vs. 15% for the control group, block stratified Odds Ratio: 0.82; 95%CI 0.34–1.96; P = 0.66) during the study period - control groups had a reduction in AE in strata 1 and 2 regardless of the availability of AMP and while there was no AE event in the intervention group, 4% of the control group had AE events in stratum 3 |
| Seol 2020[42] | Expert artificial intelligence-based natural language processing characterises childhood asthma | BMJ Open Respiratory Research | United States of America | Retrospective Cohort | Unspecified; 11.8 mean age at last follow up | Natural Language Processing | EMRs | 8196 | 36 | Assessing whether application of expert artificial intelligence (AI)- based natural language processing (NLP) algorithms for two existing asthma criteria to electronic health records of a paediatric population systematically identifies childhood asthma and its subgroups with distinctive characteristics. | - Of the 8196 subjects (51% male, 80% white), we identified 1614 (20%), NLP-PAC+/NLP-API+; 954 (12%),  NLP-PAC+ only; 105 (1%), NLP-API+ only; and 5523 (67%), NLP-PAC−/NLP-API−.  - Asthmatic children classified as NLP-PAC+/NLP-API+ showed earlier onset asthma, more Th2-high profile, poorer lung function, higher asthma exacerbation and higher risk of asthma-associated comorbidities compared with other groups - These results were consistent with those based on unsupervised cluster analysis and lab and PFT data of a random sample of study subjects - In an independent cluster analysis among asthmatics only, three clusters of subjects emerged  - Subjects in cluster A defined in the purple column and row (n=655) were characterised by a greater likelihood of persistent asthma, asthma exacerbation, pneumonia, pertussis, Pressure Equilizer (PE) tube, coeliac disease, viral and streptococcal infection, family history of asthma, eczema, allergic rhinitis, eosinophilia, no smoking during pregnancy, higher SES and spring birth  - cluster A had a disproportionately higher proportion of NLP-PAC+/NLP-API+ (82%) compared with cluster B (51%) or cluster C (55%) |
| Hee 2019[37] | Development of Machine Learning for Asthmatic and Healthy Voluntary Cough Sounds: A Proof of Concept Study | Applied Sciences | Singapore | Prospective Cohort | 0-16 years | Gaussian Mixture Model–Universal Background Model | EMRs; Health Surveys | 1192 cough sounds from 178 children | Unspecified | To develop and evaluate the sensitivity, specificity, and accuracy of a derived classification model to differentiate asthmatic coughs from normal–voluntary coughs in children compared with physicians’ diagnoses | Mel-Frequency Cepstral Coefficients (MFCCs), and Constant Q Cepstral Coefficients (CQCCs) represent perceptual cues of the audio spectrum, were extracted from the cough sounds.  Tippet Classification Accuracy (%): MFCCs - normal voluntary cough 78.91, asthma/bronchial reactivity cough 74.03, overall mean 76.24 vs. CQCCs - normal voluntary cough 78.12, asthma/bronchial reactivity cough 86.48, overall mean 82.71 vs. Fused result - normal voluntary cough 82.55, asthma/bronchial reactivity cough 84.76, overall mean 83.76  ROC Accuracy (%): MFCCs - sensitivity 78.39, specificity 75.54, AROC (95% CI) 0.8 vs. CQCCs - sensitivity 81.25, specificity 84.98, AROC (95% CI) 0.9 vs. Fused result - sensitivity 82.81, specificity 84.76, AROC (95% CI) 0.91 |
| Deliu 2020[33] | Longitudinal trajectories of severe wheeze exacerbations from infancy to school age and their association with early-life risk factors and late asthma outcomes | journal of the British Society for Allergy and Clinical Immunology | England | Prospective Cohort | 0-16 years | K-Means; Logistic Regression | EMRs; Health Surveys | 887 | Unspecified | to derive exacerbation trajectories from a population-based birth cohort with severe wheeze exacerbations confirmed in healthcare records, and examine early-life risk factors of the derived trajectories, and their asthma-related outcomes and lung function in adolescence | - Shorter duration of breastfeeding was a strong risk factor for frequent excaerbations (FE) (median weeks, FE 0 [IQR: 0-1.75] vs. IE 6 (IQR: 0-20), P < .001)  - Children in FE cluster were significantly more likely to have eczema in the first 3 years of life, but not thereafter - By age 8 years, significantly higher proportion of children in the FE cluster had doctor-diagnosed asthma (90% vs. 39%, P = .002) - The use of ICS was higher among children in FE cluster, particularly at age 3 years (80% vs. 22%, FE vs. IE, P < .001), and children in this cluster were more likely to have persistent wheeze (90% vs. 47%, P = .03) - At age 8 years, children in the FE trajectory had significantly lower FEV1 and FEV1/FVC (mean [95% CI]: FEV1% predicted, 95.6 [93.3-97.9] vs. 91.1 [80.9-101.3], P < .001; FEV1/FVC 85.1% [83.9-86.2] vs. 78.1% [72.8-83.4], P < .001), and significantly higher FeNO (ppb, mean [95% CI] 11.5 [7.8-19.5] vs. 58.5 [24.2-79.3], P < .001). AHR was higher in FE cluster, but this did not reach statistical significance (P = .08) - Children in FE cluster were significantly more likely to have current asthma in adolescence than those who wheezed, but did not have severe exacerbations in the first 8 years of life, or those in infrequent excacerbation (IF) cluster (67% FE vs. 30% IE vs. 13% WNE, P < .001).  - Lung function (ascertained by FEV1% predicted, FEV1/FVC and sRaw) was significantly diminished in the FE cluster, indicating obstructive pattern (FEV1/FVC, mean [95%CI]: 89.9% [89.3-90.5] vs. 88.1% [87.3-88.8] vs. 85.1% [83.4-86.7] vs. 74.7% [61.5-87.8], NW, WNE, IE, FE, respectively, P < .001). - FEV1 and FEV1/FVC were significantly lower from mid-school age to adolescence in the FE cluster, and declined from age 8 to age 16 years among chil-=dren in this, but not in any other group |

*Supplementary Table 2: Summary of Studies Applying Logistic Regression for Prediction of Asthma Exacerbations and Related Outcomes in Children*

| **Study** | **Study Design** | **Age Group** | **Sample Size** | **Features** | **ML Objective** | **Pertinent Results** | **Limitations** |
| --- | --- | --- | --- | --- | --- | --- | --- |
| AlSaad 2022 [30] | Retrospective Cohort | 0-18 years | 87413 | Unspecified | To predict the frequency of pediatric asthma related emergency department visits in the next 12 months | AUC-ROC: 0.81  AUC-PR: 0.69  F1: 0.56 | EHR databases lacked important variables; Data imbalance - Observational study with diagnoses or conditions that vary over time thus standard approaches for adjustment of confounding are biased |
| Bose 2021 [32] | Retrospective Cohort | 2-5 years | 9934 | 648 | To predict whether a subsequent asthma diagnosis will occur by age 10 years (individual asthma persistence), given clinical input for a child under the age of 5 years with an incident asthma diagnosis | Mean ANSA: 0.42  Median ANSA: 0.42  NPV: 0.32  Precision: 0.95  Recall: 0.81  F1 score: 0.88  Accuracy: 0.81 | Imbalanced data set; generalizability |
| Deliu 2020 [33] | Prospective Cohort | 0-16 years | 887 | Unspecified | To investigate longitudinal trajectories of severe wheeze exacerbations from infancy to school age | Two distinct trajectories of severe exacerbations from birth to mid-school age: “Infrequent exacerbations (IE)” (n = 150 [93.7%], median number of exacerbations = 1) and “Early-onset frequent exacerbations (FE)” (n = 10 [6.3%], median exacerbations number = 4). Both trajectories were associated with persistent wheeze, but had different early-life risk factors, and different asthma-related outcomes and lung function in adolescence. Shorter duration of breastfeeding was the strongest early-life risk factor for the early-onset frequent exacerbations (median weeks, FE 0 [IQR: 0-1.75] vs. IE 6 (IQR: 0-20), P < .001). | Did not have a replication population; small sample size |
| Sills 2021 [43] | Retrospective Cohort | 4-10 years | 9,069 ED visits | 13 | To predict the need for hospitalization of pediatric asthma cases using two different ML models; one with data only available at ED triage, the other adding information available one hour into the ED visit. | Triage timepoint AUC: 0.795  Triage timepoint accuracy: 0.731  Triage timepoint F1 score: 0.564  60 minutes post arrival AUC: 0.823  60 minutes post arrival accuracy: 0.753  60 minutes post arrival F1 score: 0.618 | Lack of valuable patient clinical information, such as weight or heart rate at triage; may include error and noises from data collection; exclusion of patients who received a late medication delivery |
| Hogan 2022 [38] | Retrospective Cohort | 5-18 years | 18489 | 18 | To predict whether a child has an asthma readmission within 180 days from hospitalization discharge | Overall model AUC: 0.592 | Lack of longitudinal outcome data; lack of risk factors related to asthma readmissions in data set |
| Bhardwaj 2023 [31] | Retrospective Cohort | 1-5 years | 205 | 93 | To classify two forms of asthma in preschool children (predominantly allergic asthma and non-allergic asthma) | Accuracy: 76.2%  Precision: 0.81  TPR 0.74  TNR: 0.78  F1: 0.79  ROC-AUC: 0.80 | Small sample size; due to a high number of missing data points, features were eliminated |
| Gorham 2023 [35] | Retrospective Cohort | 2-18 years | 26008 | 8 | To predict which patients will visit the ED due to asthma exacerbation within one year of a primary care encounter | AUROC during model training: 0.768  AUROC against 2018 NCH internal validation: 0.769  AUROC against EU population without local retraining: 0.684  AUROC against EU population with local retraining: 0.737 | Data limited to 2 data centres; differences in exclusion at validation site; did not add novel predictors during the external validation |

*Supplementary Table 3: Summary of Studies Applying Random Forests for Prediction of Asthma Exacerbations and Related Outcomes in Children*

| **Study** | **Study Design** | **Age Group** | **Sample Size** | **Features** | **ML Objective** | **Pertinent Results** | **Limitations** |
| --- | --- | --- | --- | --- | --- | --- | --- |
| Krautenbacher  2019 [40] | Prospective Cohort | 4-14 years | 260 | 7 modalities | To predict different childhood asthma phenotypes (healthy children, mild‐to‐moderate allergic asthmatics, and nonallergic asthmatic) based off an assembled questionnaire, diagnostic, genotype, microarray, RT‐qPCR, flow cytometry, and cytokine data as inputs | AUC for performed prediction on single modalities separately: 0.66  Overall AUC for complete observations including annotated genes: 0.7  Overall AUC for complete observations including non-annotated genes: 0.7 | Small sample size; lack of additional clinical phenotypes, such as distinct wheeze phenotypes was not used |
| Bose  2021 [32] | Retrospective Cohort | 2-5 years | 9934 | 648 | To predict whether a subsequent asthma diagnosis will occur by age 10 years (individual asthma persistence), given clinical input for a child under the age of 5 years with an incident asthma diagnosis | Mean ANSA: 0.42  Median ANSA: 0.44  NPV: 0.33  Precision: 0.95  Recall: 0.82  F1 score: 0.88  Accuracy: 0.88 | Imbalanced data set; generalizability |
| Deng  2021 [34] | Prospective Cohort | 8-14 years | 74 | 84 | To use machine-learning methods to evaluate how indoor environmental conditions at home and school contribute to asthma and allergy-related symptoms | The top contributing factors identified for asthma were family rhinitis history, plant pollen trigger, bedroom carpet, environmental tobacco smoke (ETS) trigger symptom, and ETS exposure  For allergy-related symptoms, plant pollen trigger, higher paternal education, bedroom carpet, family rhinitis history, and higher maternal education were the strongest contributing factors primary heating with hot water radiator was negatively associated with asthma symptoms | Recall/reporting bias; potential for misclassification of asthma and allergy |
| Sills 2021 [43] | Retrospective Cohort | 4-10 years | 9,069 ED visits | 13 | To predict the need for hospitalization of pediatric asthma cases using two different ML models; one with data only available at ED triage, the other adding information available one hour into the ED visit. | Triage timepoint AUC: 0.831  Triage timepoint accuracy: 0.777  Triage timepoint F1 score: 0.635  60 minutes post arrival AUC: 0.886  60 minutes post arrival accuracy: 0.795  60 minutes post arrival F1 score: 0.689 | Lack of valuable patient clinical information, such as weight or heart rate at triage; may include error and noises from data collection; exclusion of patients who received a late medication delivery |
| Hurst  2021 [39] | Retrospective Cohort | 5-18 years | 5982 | 12 | To predict whether a child will have an asthma related exacerbation in the forthcoming 30-180 days. | AUC of overall model of exacerbation in 30 days: 0.757  AUC of overall model of exacerbation in 90 days: 0.747  AUC of overall model of exacerbation in 180 days: 0.729 | Generalizability; did not account for all variables such as indoor environment and viral exposures |
| Bhardwaj 2023 [31] | Retrospective Cohort | 1-5 years | 205 | 93 | To classify two forms of asthma in preschool children (predominantly allergic asthma and non-allergic asthma) | Accuracy: 67.3%  Precision: 0.71  TPR 0.59  TNR: 0.72  F1: 0.72  ROC-AUC: 0.72 | Small sample size; due to a high number of missing data points, features were eliminated |

*Supplementary Table 4: Summary of Studies Applying Gradient Boosting for Prediction of Asthma Exacerbations and Related Outcomes in Children*

| **Study** | **Study Design** | **Age Group** | **Sample Size** | **Features** | **ML Objective** | **Boosting Technique** | **Pertinent Results** | **Limitations** |
| --- | --- | --- | --- | --- | --- | --- | --- | --- |
| Krautenbacher  2019 [40] | Prospective Cohort | 4-14 years | 260 | 7 modalities | To predict different childhood asthma phenotypes (healthy children, mild‐to‐moderate allergic asthmatics, and nonallergic asthmatic) based off an assembled questionnaire, diagnostic, genotype, microarray, RT‐qPCR, flow cytometry, and cytokine data as inputs | Stochastic gradient boosting | AUC for performed prediction on single modalities separately: 0.54  Overall AUC for complete observations including annotated genes: 0.77  Overall AUC for complete observations including non-annotated genes: 0.81 | Small sample size; lack of additional clinical phenotypes, such as distinct wheeze phenotypes was not used |
| Bose  2021 [32] | Retrospective Cohort | 2-5 years | 9934 | 648 | To compare different ML techniques that can predict the occurrence of early childhood asthma persistence | XGB | Mean ANSA: 0.43  Median ANSA: 0.43  NPV: 0.34  Precision: 0.95  Recall: 0.82  F1 score: 0.88  Accuracy: 0.81 | Imbalanced data set; generalizability |
| Hurst  2021 [39] | Retrospective Cohort | 5-18 years | 5982 | 12 | To predict whether a child will have an asthma related exacerbation in the forthcoming 30-180 days. | XGB | AUC of overall model of exacerbation in 30 days: 0.761  AUC of overall model of exacerbation in 90 days: 0.752  AUC of overall model of exacerbation in 180 days: 0.077 | Generalizability; did not account for all variables such as indoor environment and viral exposures |
| Bhardwaj 2023 [31] | Retrospective Cohort | 1-5 years | 205 | 93 | To classify two forms of asthma in preschool children (predominantly allergic asthma and non-allergic asthma) | XGB | Accuracy: 66.3%  Precision: 0.69  TPR 0.54  TNR: 0.75  F1: 0.71  ROC-AUC: 0.71 | Small sample size; due to a high number of missing data points, features were eliminated |

*Supplementary Table 5: Summary of Studies Applying Artificial Neural Networks (ANN) for Prediction of Asthma Exacerbations and Related Outcomes in Children*

| **Study** | **Study Design** | **Age Group** | **Sample Size** | **Features** | **ML Objective** | **Pertinent Results** | **Limitations** |
| --- | --- | --- | --- | --- | --- | --- | --- |
| AlSaad 2022 [30] | Retrospective Cohort | 0-18 years | 87413 | Unspecified | To predict the frequency of pediatric asthma related emergency department visits in the next 12 months | RETAIN:  AUC-ROC (micro, macro): 0.85, 0.72  AUC-PR (micro, macro): 0.74, 0.44  F1 (micro, macro): 0.66, 0.60  BiLSTM:  AUC-ROC (micro, macro): 0.85, 0.71  AUC-PR (micro, macro): 0.74, 0.48  F1 (micro, macro): 0.67, 0.60  BiGRU:  AUC-ROC (micro, macro): 0.85, 0.71  AUC-PR (micro, macro): 0.74, 0.46  F1 (micro, macro): 0.66, 0.61 | EHR databases lacked important variables; Data imbalance - Observational study with diagnoses or conditions that vary over time thus standard approaches for adjustment of confounding are biased |
| Messinger 2019 [41] | Retrospective Cohort | 2-18 years | 128 | 3 | To create pediatric‐automated asthma respiratory score (pARS) by using the manual pediatric asthma score (PAS) as the clinical care standard | MAE of cascaded ANN with eight hidden layers trained with the balanced group: 1.21 | Incomplete data; generalizability; missing variables such as acute bronchodilator use |
| Hogan 2022 [38] | Retrospective Cohort | 5-18 years | 18489 | 18 | To predict whether a child has an asthma readmission within 180 days from hospitalization discharge | AUC in training set: 0.637  AUC in validation set: 0.636 | Lack of longitudinal outcome data; lack of risk factors related to asthma readmissions in data set |

*Supplementary Table 6: Summary of Studies Applying Decision Tree for Prediction of Asthma Related Symptoms*

| **Study** | **Study Design** | **Age Group** | **Sample Size** | **Features** | **ML Objective** | **Pertinent Results** | **Limitations** |
| --- | --- | --- | --- | --- | --- | --- | --- |
| Deng  2021 [34] | Prospective Cohort | 8-14 years | 74 | 84 | To use machine-learning methods to evaluate how indoor environmental conditions at home and school contribute to asthma and allergy-related symptoms | Asthma symptoms:  AUC for internal validation: 0.797  AUC for 10-fold cross-validation: 0.753  AUC for external validation: 0.812  Allergy-related symptoms:  AUC for internal validation: 0.819  AUC for 10-fold cross-validation: 0.790  AUC for external validation: 0.829 | Recall/reporting bias; potential for misclassification of asthma and allergy |
| Habukawa 2020 [36] | Prospective Cohort | 0-13 years | 813 recorded sounds | 5 | Automatic wheeze sound recognition | PPV 90.3%  NPPV 100%  Sensitivity (wheeze sounds): 100%  Sensitivity (non-wheeze sounds): 95.7% | Intensity of wheezes varies among children; For lung sounds that were recorded in a noisy clinic, more rigorous post-processing is required; generalizability between clinical settings and home |

*Supplementary Table 7: Summary of Studies Applying Language Processing for Prediction of Asthma Related Symptoms*

| **Study** | **Study Design** | **Age Group** | **Sample Size** | **Features** | **ML Objective** | **Pertinent Results** | **Limitations** |
| --- | --- | --- | --- | --- | --- | --- | --- |
| Seol 2020 [42] | Cross-sectional analysis within a retrospective birth cohort | 0-15 years old | 8196 children | N/A | If natural language processing can use EMR data to identify childhood asthma | n=8196 children, 1679 (21%) had obtained an asthma diagnosis at a mean age of diagnosis at 4.9 (±3.8) years) compared to both algorithms with a mean age at asthma index date = 3.9 (±3.8) years). Children classified as positive under both asthma criteria showed earlier onset asthma (p <0.001), and higher exacerbation rates (p <0.001). | Laboratory, and lung function data was not available for all study subjects |
| Seol 2021 [25] | Randomized Control Trial | pediatric (specific age not specified) | 184 | N/A | To determine if a machine-learning-based predictive analytics for future asthma exacerbation (AE) can reduce AE frequency in children | Proportion of children with AE in both groups decreased from the baseline (P=0.042), there was no difference in AE frequency between the two groups(12% for the intervention group vs.15% for the control group, Odds Ratio:0.82; 95%CI 0.374–1.96; P= 0.626). A-GPS intervention significantly reduced time for reviewing EHRs for asthma management of each participant (median:3.5min,IQR:2–5),compared to usua lcare without A-GPS (median:11.3min, IQR:6.3–15);p<0.001). | Generalizability; did not include lung function measures or medications in defining persistent asthma and study outcomes |

*Supplementary Table 8: Summary of Studies Applying Gaussian Mixture Model for Prediction of Asthma Related Symptoms*

| **Study** | **Study Design** | **Age Group** | **Sample Size** | **Features** | **ML Objective** | **Pertinent Results** | **Limitations** |
| --- | --- | --- | --- | --- | --- | --- | --- |
| Hee 2019 [37] | Prospective Cohort Pilot Study | Under 16 years old | 89 children | N/A | Evaluation of the sensitivity and specificity of the classification model to differentiate asthmatic coughs from normal–voluntary coughs in children compared with physicians’ diagnoses | Sensitively (82.81%),  Specificity (84.76 %) (AROC; 95%CI 0.91; 0.89-0.93). | cough sounds from asthmatic children with concurrent  respiratory conditions were excluded |
